# The Dis1/Stu2/XMAP215 Family Gene *Fg*Stu2 Is Involved in Vegetative Growth, Morphology, Sexual and Asexual Reproduction, Pathogenicity and DON Production of *Fusarium graminearum*

**DOI:** 10.3389/fmicb.2020.545015

**Published:** 2020-11-20

**Authors:** Yuanye Zhu, Yuanshuai Zhang, Na Liu, Weichao Ren, Yiping Hou, Yabing Duan, Xiushi Song, Mingguo Zhou

**Affiliations:** ^1^College of Plant Protection, Nanjing Agricultural University, Nanjing, China; ^2^College of Plant Health and Medicine, Qingdao Agricultural University, Qingdao, China

**Keywords:** *Fusarium graminearum*, *Fg*Stu2, microtubule, vegetative growth, morphology, sexual and asexual reproduction, DON production

## Abstract

The conserved Dis1/Stu2/XMAP215 microtubule association proteins (MAPs) family plays an important role in microtubule dynamics, nucleation, and kinetochore-microtubule attachments. However, function of Dis1/Stu2/XMAP215 homolog in plant pathogenic fungi has not been determined. Here, we identified and investigated the Dis1/Stu2/XMAP215 homolog (FGSG_10528) in *Fusarium graminearum* (*Fg*Stu2p). Co-localization experiment and co-immunoprecipitation (Co-IP) assay demonstrated that *Fg*Stu2p is a microtubule associated protein. Besides, *Fg*Stu2 could also interact with *Fg*γ-tubulin and presumed *Fg*Ndc80, which suggested that the *Fg*Stu2 gene might associate with microtubule nucleation and kinetochore-microtubule attachments like Dis1/Stu2/XMAP215 homologs in other species. Moreover, the *Fg*Stu2 promoter replacement mutants (*Fg*Stu2-Si mutants) produced twisted hyphae and decreased growth rate. Microscope examination further showed that the microtubule polymerization was reduced in *Fg*Stu2-Si mutants, which could account for the aberrant morphology. Although the microtubule polymerization was affected in *Fg*Stu2-Si mutants, the *Fg*Stu2-Si mutants didn’t show highly increased sensitivity to anti-microtubule fungicide carbendazim (methyl benzimidazol-2-ylcarbamate [MBC]). In addition, the *Fg*Stu2-Si mutants exhibited curved conidia, decreased number of conidial production, blocked ability of perithecia production, decreased pathogenicity and deoxynivalenol (DON) production. Taken together, these results indicate that the *Fg*Stu2 gene plays a crucial role in vegetative growth, morphology, sexual reproduction, asexual reproduction, virulence and deoxynivalenol (DON) production of *F. graminearum*, which brings new insights into the functions of Dis1/Stu2/XMAP215 homolog in plant pathogenic fungi.

## Introduction

Microtubules are hollow cylindrical polymers assembled from α/β-tubulin heterodimers that have a specific behavior: microtubules switch between growing or shrinking states, a property known as dynamic instability, which is vital to many intracellular activities such as mitosis, material transportation and cell morphology (Nogales, 2001; Tischfield et al., 2010; Aher and Akhmanova, 2018). Individual microtubule has two different tips with distinct conformations, the minus end and the plus end. The minus end is the nucleation site of microtubule. While the plus end is the growing end which could recruit or disassociate α/β-tubulin heterodimers (Schuyler and Pellman, 2001).

Microtubule association proteins (MAPs) are a great kind of proteins that could attach to microtubule or tubulin and regulate microtubule dynamics (Walczak, 2000). One of the most important MAPs families is the Dis1/Stu2/XMAP215 family which was first identified in *Xenopus laevis* (Gard and Kirschner, 1987). In the present, the Dis1/Stu2/XMAP215 family has been widely studied in *X. laevis* (XMAP215), Human (ch-TOG, human XMAP215 homolog), budding yeast (Stu2, budding yeast XMAP215 homolog), and fission yeast (Dis1, fission yeast XMAP215 homolog) (Al-Bassam et al., 2006; Al-Bassam and Chang, 2011). The Dis1/Stu2/XMAP215 family was famous as they have 2-5 conserved tumor-overexpressed gene (TOG) domains at amino-terminus and a microtubule binding domain at carboxy-terminus (Thawani et al., 2018). TOG domain could bind to α/β-tubulin heterodimers and is essential for microtubule polymerization activity of Dis/Stu2/XMAP215 family (Ayaz et al., 2012; Geyer et al., 2018). Previous studies showed that knockdown of these proteins often leads to small or abnormally organized spindles and short astral microtubules (Al-Bassam and Chang, 2011). Moreover, the Dis1/Stu2/XMAP215 family could also act together with other MAPs such as end binding 1 (EB1) to regulate microtubule dynamics (Zanic et al., 2013). At kinetochores, the Dis1/stu2/XMAP215 family associates with the Ndc80 kinetochore complex and regulates the kinetochore-microtubule attachment which is vital for cytokinesis (Hsu and Toda, 2011; Miller et al., 2016). Recent studies showed that the Dis1/stu2/XMAP215 family is a microtubule nucleation factor that functions synergistically with the γ-tubulin ring complex (Thawani et al., 2018). In addition, in budding yeast, Stu2p promote oligomerization of the γ-tubulin ring complex (γ-TuRC) and cytoplasmic microtubule nucleation via interaction with Spc72 (Chen et al., 1998). Taken together, Dis/Stu2/XMAP215 family is essential for microtubule polymerization and function of kinetochore and disrupt these gene is fatal for the cell. Although Dis/Stu2/XMAP215 family has been well studied in human, yeast and *X. laevis*, its role in plant pathogenic fungi has not been determined. Therefore, in this study, we investigated the functions of Dis/Stu2/XMAP215 family homolog in *Fusarium graminearum* (*Fg*Stu2).

*Fusarium graminearum* (teleomorph: Gibberella zeae), a universal plant pathogenic fungi which causes the *Fusarium* head blight (FHB), a destructive disease in cereal crops (Bai and Shaner, 2004). In addition to give rise to serious yield loss, FHB can also contaminate grain by and mycotoxin such as deoxynivalenol (DON) and zearalenone (ZEA), which posed severe threaten to human and livestock health (Forsyth et al., 1977; Chen et al., 2019). DON is the most widely detected mycotoxin in cereal corps with a 56% average incidence rate worldwide compared with 14% of zearalenone and other mycotoxin (Lee and Ryu, 2017). Due to the highest occurrence and toxicity, DON has gained more attention and has been widely studied (Lee and Ryu, 2017; Chen et al., 2019). Although many genes of *F. graminearum* have been demonstrated to regulate the DON production, the role of microtubule-associated proteins such as the Dis/Stu2/XMAP215 family in DON production has not been widely characterized (Chen et al., 2019). Therefore, we are interested in the functional role of *Fg*Stu2 gene in DON production.

In the present, control of FHB is mainly depended on chemical fungicides due to lack of fully resistant cultivars (Blandino et al., 2012). One of the most widely used fungicides for control FHB is carbendazim (methyl benzimidazol-2-ylcarbamate [MBC]), which binds to tubulin heterodimers and disrupt microtubule dynamics (Davidse, 1986; Chen et al., 2009). Previous studies have shown that *F. graminearum* contains two β-tubulin genes: β_1_- and β_2_-tubulin, and mutations such as F167Y, E198K, and F200Y in β_2_-tubulin gene confer resistance to MBC (Chen et al., 2009; Qiu et al., 2012). In this study, we also investigated the relationship between *Fg*Stu2 and *Fg*β1- and *Fg*β2-tubulin and the role of *Fg*Stu2 in regulation of MBC sensitivity. By the means of promoter substitution, Co-immunoprecipitation (Co-IP), EGFP (Enhanced green fluorescent protein)-tagging, and RFP (Red fluorescent protein)-tagging, we revealed the functional importance of *Fg*Stu2 gene in *F. graminearum.* Our results will provide information about the functional role of *Fg*Stu2 gene and its relationship with tubulin.

## Materials and Methods

### Sequence Alignment and Analysis

**FIGURE 1:**
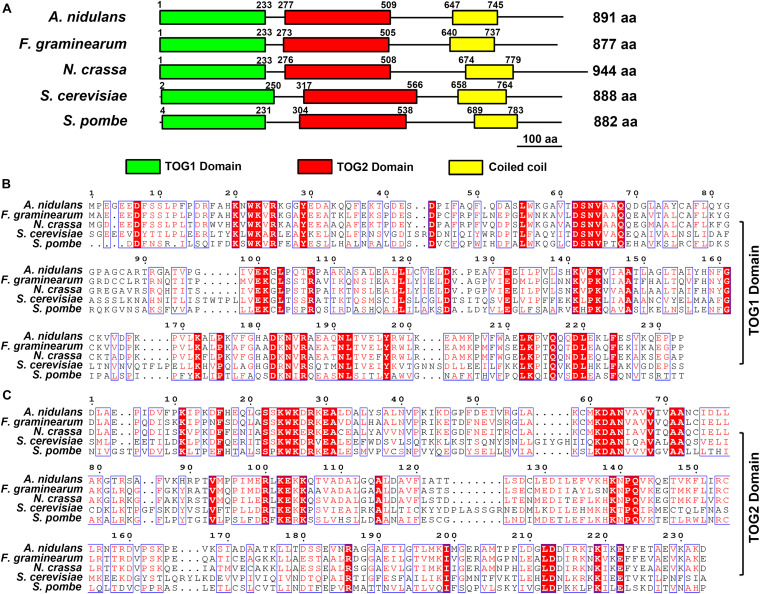
Domain analysis of Stu2 homologs and sequences alignment. **(A)** Domain analysis of Dis1/Stu2/XMAP215 homologs in *Aaspergillus nidulans* (Protein ID: XP_663125.1), *Fusarium graminearum* (Protein ID: XP_011319522.1), *Neurospora crassa* (Protein ID: XP_956946.3), *Saccharomyces cerevisiae* (Protein ID: NP_013146.1) and *Schizosaccharomyces pombe* (Protein ID: NP_587785.1). The Domain prediction was conducted by the online service of the Simple Modular Architecture Research Tool (SMART, https://smart.embl.de). **(B)** Sequences alignment of TOG1 domain. Multiple sequences alignment was performed using the online service (www.genome.jp/tools-bin/clustalw). The alignment images were generated by the online service of ESPript 3.0 (espript.ibcp.fr/ESPript/cgi-bin/ ESPript.cgi). **(C)** Sequences alignment of TOG2 domain. The red background indicates the same amino acid residues.

We used the amino acid sequences of *Saccharomyces cerevisiae* Stu2 (Gene bank ID: 856736) and *S. pombe* Dis1 (Gene bank ID: 2539505) to find the Dis1/Stu2/XMAP215 homologs in *F. graminearum* (*Fg*Stu2 gene bank ID: 23557431). The protein blast was carried out by online blast services of the National Center for Biotechnology information (NCBI^1^) and the Ensembl Fungi^2^. The presumed *Fg*Ndc80 gene (FGSG_09262, gene bank ID: 23556224) was also identified by online blast services of the NCBI. The phylogenetic analysis (phylogenetic tree) of Dis1/Stu2/XMAP215 homologs of *Aspergillus nidulans* (Protein ID: XP_663125.1), *F. graminearum* (Protein ID: XP_011319522.1), *Neurospora crassa* (Protein ID: XP_956946.3), *S. cerevisiae* (Protein ID: NP_013146.1) and *S. pombe* (Protein ID: NP_587785.1) was finished by the Molecular Evolutionary Genetics Analysis software (MEGA, version: 7.0) (Supplementary Figure 1). The domain prediction was conducted by the online service of Simple Modular Architecture Research Tool (SMART^3^) (Letunic et al., 2014). Multiple sequences alignments of tumor-overexpressed gene (TOG) domains and terminal areas in Dis/Stu2/XMAP215 homologs of *A. nidulans* (Protein ID: XP_663125.1), *F. graminearum* (Protein ID: XP_011319522.1), *N. crassa* (Protein ID: XP_956946.3), *S. cerevisiae* (Protein ID: NP_013146.1), and *S. pombe* (Protein ID: NP_587785.1) were performed using the online service^4^ (Figures 1B,C and Supplementary Figure 2). The amino acids sequences alignment of presumed *Fg*Ndc80 (protein ID: XP_011328506.1) and budding yeast Ndc80 (protein ID: NP_012122.3) was performed using the same online service (Supplementary Figure 3). The alignment images were generated by the online service of ESPript 3.0^5^ (Robert and Gouet, 2014).

### Strains, Cultural Condition, and Fungicides

All strains used in this study are listed in Table 1. The PH-1 strain is the standard strain of *F. graminearum* which was completely sequenced in Cuomo et al. (2007). In the recent years, PH-1 strain has become a model strain in plant pathology and fungal genetics (Liu et al., 2017; Chen et al., 2019; Jiang et al., 2020). More importantly, the genetic transformation and fluorescence labeling technologies of PH-1 are now perfect and convenient. Taken together, we would like to use PH-1 strain to study the functional role of Dis1/Stu2/XMAP215 homolog in plant pathogenic fungi and the results would be more representative than the other wild-type strain. Conidia and mycelia were produced in mung bean liquid (MBL) medium and yeast extract peptone dextrose (YEPD) medium at 25°C respectively. For colonial morphology, strains were grown on complete medium (CM) and minimal medium (MM) plants at 25°C (Gu et al., 2015). For sensitivity assays, all strains were grown on potato dextrose agar (PDA) containing carbendazim (MBC) at 25°C. DON mycotoxin was produced in trichothecene biosynthesis induction (TBI) medium (Gardiner et al., 2009). MBC were dissolved in 0.1 M HCl at 10 mg/ml as stock solutions (Chen et al., 2009).

**TABLE 1:** Strains used in this study.

Strains and mutants	Genotype	References
PH-1	*Fusarium graminearum* wild-type strain	Cuomo et al., 2007
*Fg*β2F167Y	*Fg*β2-tubulin F167Y mutant generated from PH-1	This study
*Fg*Stu2-Si-1	Promoter replacement mutant of *Fg*Stu2 gene generated from PH-1	This study
*Fg*Stu2-Si-2	Promoter replacement mutant of *Fg*Stu2 gene generated from PH-1	This study
*Fg*β2F167Y-Stu2-Si	Promoter replacement mutant of *Fg*Stu2 gene generated from *Fg*β2F167Y	This study
*Fg*Stu2-3 × FLAG	*Fg*Stu2-3 × FLAG generated from PH-1	This study
*Fg*β1-tubulin-EGFP	*Fg*β1tubulin-EGFP generated from PH-1	This study
*Fg*β2-tubulin-EGFP	*Fg*β2tubulin-EGFP generated from PH-1	This study
*Fg*β1EGFP-Stu2-3 × FLAG	*Fg*Stu2-3 × FLAG generated from *Fg*β1-tubulin-EGFP	This study
*Fg*β2EGFP-Stu2-3 × FLAG	*Fg*Stu2-3 × FLAG generated from *Fg*β2-tubulin-EGFP	This study
*Fg*Stu2-Si-β1EGFP	*Fg*β1-tubulin-EGFP generated from *Fg*Stu2 promoter replacement mutant	This study
*Fg*Stu2-Si-β2EGFP	*Fg*β2-tubulin-EGFP generated from *Fg*Stu2 promoter replacement mutant	This study
*Fg*β2EGFP-Stu2mRFP	*Fg*Stu2-mRFP mutant generated from *Fg*β2EGFP	This study
*Fg*γ-tubulin-EGFP	*Fg*γtubulin-EGFP mutant generated from PH-1	This study
*Fg*Ndc80-EGFP	*Fg*Ndc80-EGFP mutant generated from PH-1	This study
*Fg*γEGFP-Stu2-3 × FLAG	*Fg*Stu2-3 × FLAG generated from *Fg*γtubulin-EGFP	This study
*Fg*Ndc80-EGFP-Stu2-3 × FLAG	*Fg*Stu2-3 × FLAG generated from *Fg*Ndc80-EGFP	This study
PH-1-Tri1-EGFP	*Fg*Tri1-EGFP strain generated from PH-1	This study
*Fg*Stu2-Si-Tri1-EGFP	*Fg*Tri1-EGFP strain generated from *Fg*Stu2-Si mutant	This study

### Construction of EGFP-, mRFP-, 3 × FLAG-Tagging Mutants

**FIGURE 2:**
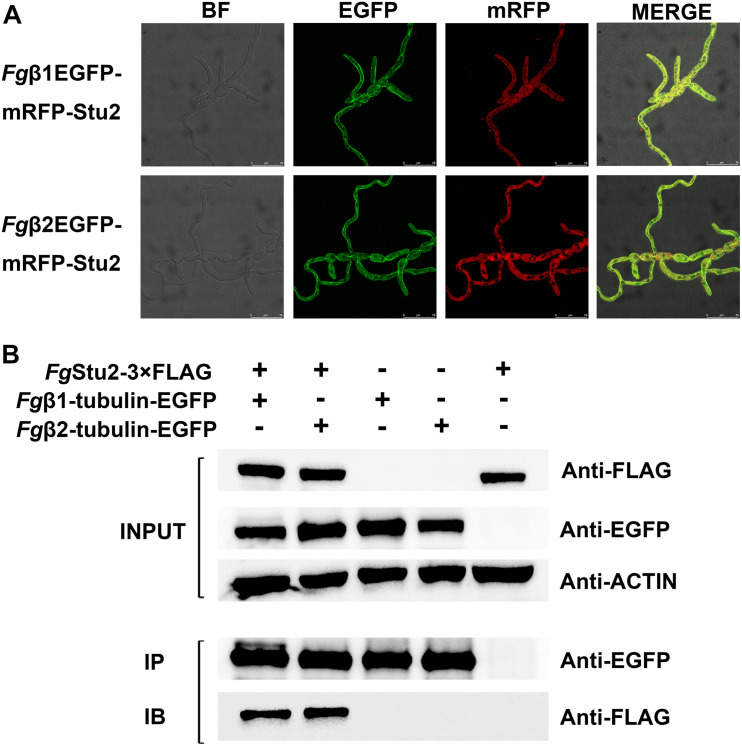
Microtubule-associated properties of *Fg*Stu2p **(A)** Co-localization of RFP-*Fg*Stu2 and *Fg*β-tubulin-EGFP. Fresh spores (10^5^) of all double-labeling mutants were first incubated into 20 ml of yeast extract-peptone-dextrose medium (YEPD) on an orbital shaker (175 rpm) at 25°C to germinate for 10 h and then spotted 5 μl of culture onto the slides for micro examination. Bar = 25 μm. **(B)** Co-immunoprecipitation (Co-IP) analysis of the interaction of *Fg*Stu2p and *Fg*β1/β2-tubulin. The INPUT (total protein) indicates that the double labeling strains (*Fg*β1EGFP-Stu2-3 × FLAG and *Fg*β2EGFP-Stu2-3 × FLAG) and single labeling strains (*Fg*Stu2-3 × FLAG, *Fg*β1-tubulin-EGFP, *Fg*β2-tubulin-EGFP) were successfully labeled with FLAG and EGFP tags respectively; the protein samples were also detected with anti-actin antibody as a reference; the IP (Immunoprecipitation, polyclonal anti-EGFP rabbit antibody) indicates the *Fg*β1-tubulin-EGFP and *Fg*β2-tubulin-EGFP were precipitated by Protein-G magnetic beads which had been incubated with polyclonal anti-EGFP rabbit antibody; the IB (Immune blotting, monoclonal anti-FLAG mouse antibody) indicates that the *Fg*Stu2-3 × FLAG was pulled down by the *Fg*β1-tubulin-EGFP or *Fg*β2-tubulin-EGFP.

**FIGURE 3:**
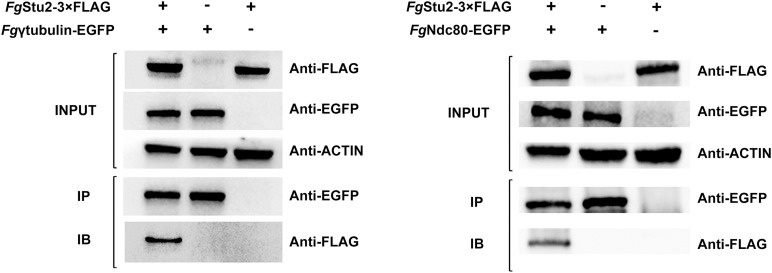
Co-immunoprecipitation (Co-IP) analysis of interactions of *Fg*Stu2p-*Fg*γ-tubulin and *Fg*Stu2p-*Fg*Ndc80p. The INPUT (total protein) indicates that the double labeling strains (*Fg*γEGFP-Stu2-3 × FLAG and *Fg*Ndc80-EGFP-Stu2-3 × FLAG) and single labeling strains (*Fg*Stu2-3 × FLAG, *Fg*γ-tubulin-EGFP, *Fg*Ndc80-EGFP) were successfully labeled with FLAG and EGFP tags respectively; the IP (Immunoprecipitation, polyclonal anti-EGFP rabbit antibody) indicates the *Fg*γ-tubulin-EGFP, *Fg*Ndc80-EGFP were precipitated by Protein-G magnetic beads which had been incubated with polyclonal anti-EGFP antibody; the IB (Immune blotting, monoclonal anti-FLAG mouse antibody) indicates that the *Fg*Stu2-3 × FLAG was pulled down by the *Fg*γ-tubulin-EGFP, *Fg*Ndc80-EGFP.

The *Fg*β_1_-tubulin-EGFP, *Fg*β_2_-tubulin-EGFP single labeling strains (Table 1) were stored in our lab. The *FgStu2*-3 × FLAG strain was constructed using homologous recombination strategy (Supplementary Figure 4), first a 1.0 kb fragment of *FgStu2* 3′-terminus coding sequence was amplified from genomic DNA of PH-1 by primer containing 1 × FLAG-tag sequence. This fragment was used as a template for another two rounds of PCR to add 2 × FLAG-tag. Second, a 1.2 kb geneticin (G418) fragment was amplified from pNEO. Third, a 1.0 kb *FgStu2* downstream fragment was amplified from genomic DNA of PH-1. Finally, the three fragments were fused by DJ-PCR and used as a template for amplify the *FgStu2*-3 × FLAG vector. Transformates were identified by PCR, sequencing and western blot (Figure 2 and Supplementary Figure 4). For constructing the *Fg*Stu2-Si-β1EGFP and *Fg*Stu2-Si-β2EGFP strains, we cloned the *Fg*β1-tubulin and *Fg*β2-tubulin coding sequences (CDS) into PYF11 plasmid and then transformed these plasmids into protoplasts of *Fg*Stu2-Si mutant to generate the *Fg*Stu2-Si-β1EGFP and *Fg*Stu2-Si-β2EGFP strains (Bruno et al., 2004). For constructing the *Fg*β2EGFP-Stu2mRFP strain, we cloned RFP-Stu2 DNA fragment into PYF11 plasmid and then transformed this plasmid into protoplasts of *Fg*β_2_-tubulin-EGFP strain to generate the *Fg*β2EGFP-Stu2mRFP strain. The *Fg*γ-tubulin-EGFP and *Fg*Ndc80-EGFP strains were constructed using homologous recombination strategy. First, a 1.0 kb 3′-terminal CDS of *Fg*γ-tubulin (Gene bank ID: 23556916) and presumed *Fg*Ndc80 gene (Gene bank ID: 23556224) was amplify from the genomic DNA of wild-type strain PH-1; a 0.7 kb EGFP CDS and 1.7 kb hygromycin resistance gene (HPH) fragment were amplified from PYF11 plasmid and pNEO plasmid respectively, and then fused the two fragments by double-joint PCR to generate a 2.4 kb GFP-HPH fragment; a 1.0 kb downstream fragment of *Fg*γ-tubulin and presumed *Fg*Ndc80 gene was amplified from the genomic DNA of PH-1. Finally, fused the three fragments by double-joint PCR to generate the *Fg*γ-tubulin-EGFP and *Fg*Ndc80-EGFP vectors and then transferred the vectors into protoplasts of PH-1. The transformates were identified by PCR, sequencing, and western blot (Figure 3 and Supplementary Figure 5). Protoplasts transformations were performed as previously described (Zheng et al., 2014).

### Co-immunoprecipitation (Co-IP) Assays

Strains containing EGFP or 3 × FLAG tag were inocubated into MBL medium for 3 days. After that, fresh spores were inoculated into 100 ml YEPD medium for 24 h at 25°C with agitation (175 rpm) and then fresh mycelia were collected and ready for use. For total protein extraction, fresh dried pressed mycelia were quickly frozen in liquid nitrogen and ground into powder. For each strain, 0.15 g frozen mycelial powder was suspended in 1.5 mL of extraction buffer (20 mM Tris, 200 mM NaCl, 0.1% Triton X-100, 1 × protease inhibitor cocktail [20124ES10, YiSheng Biotechnology Inc., Shanghai, China], PH = 7.5). After incubation on ice for 30 min, cell debris was spun down at 12000*g* for 20 min at 4°C, and the clear supernatant was transferred to a new tube. The Co-IP assay was conducted follow the standard immunoprecipitation protocol of protein G magnetic beads (SureBeads^TM^, Bio-Rad Laboratories, lnc).

### Western Blotting Assays

Protein samples was added 5 × Loading buffer and boiled for 5 min before loaded into Sodium dodecyl sulfate-polyacrylamide gel. Protein separated by Sodium dodecyl sulfate-polyacrylamide gel electrophoresis (SDS-PAGE) was transferred to polyvinylidene fluoride membrane (PVDF) with a Bio-Rad electroblotting device. After that, the PVDF membrane was incubated with 5% defatted milk (5 g non-fat dry milk diluted into 100 ml TBST buffer [10 mM Tris, 100 mM NaCl, 0.1% Tween 20, PH = 7.4]) and blocking for 1 h at 25°C. After Blocking, antibody (polyclonal Anti-EGFP Rabbit antibody, monoclonal Anti-FLAG mouse antibody and monoclonal Anti-Actin antibody were purchased form Zhengneng Biotechnology, Inc., Chengdu, China) was added into the defatted milk. After incubation at 25°C for 2 h, the PVDF membrane was washed with TBST buffer for 5 min with 3 buffer changes and then incubated for 1 h using horse radish peroxidase (HRP)-conjugated secondary antibody diluted into 5% defatted milk. Finally, the PVDF membrane was washed three times by TBST and ready for blotting.

### Promoter Replacement Strategy

**FIGURE 4:**
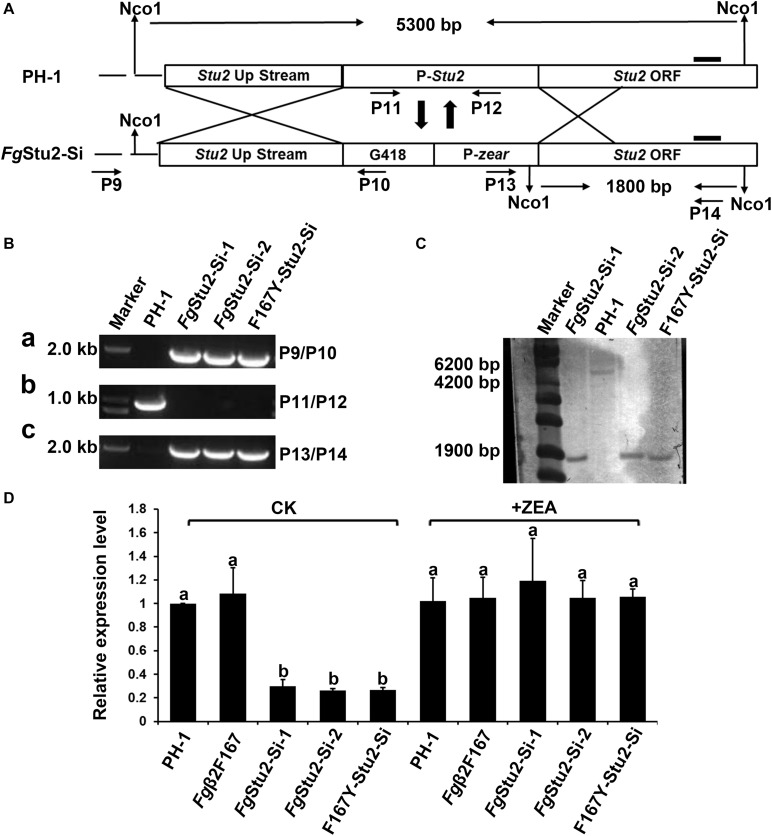
Promoter replacement strategy and identification of promoter replacement mutant of *Fg*Stu2 gene. **(A)** Schematic of promoter replacement of *Fg*Stu2 gene. **(B)** Polymerase chain reaction (PCR) identification of *Fg*Stu2 promoter replacement mutants. **(a)** a 1.9 kb fragment was amplified by primers P9/P10 indicates G418-Pzear fragment integrated at the 3′-terminal of up-stream non-coding sequence of FgStu2 gene. **(b)** a 0.9 kb fragment could not amplified by primers P11/P12 indicates original promoter of *Fg*Stu2 has been substituted with G418-Pzear. **(C)** a 1.8 kb fragment was amplified by primers P13/P14 indicates that G418-Pzear integrated at the 5′-terminal of *Fg*Stu2 coding sequence (CDS). **(C)** Southern blot identification of *Fg*Stu2 promoter replacement mutants. **(D)** Real-time quantitative PCR identification of *Fg*Stu2 promoter replacement mutants.

To study the functional role of *Fg*Stu2 gene, we substituted original *Fg*Stu2 promoter with an inducible promoter Pzear (Promoter of the FGSG_04581.3 gene) to generate *Fg*Stu2 silent mutant (*Fg*Stu-Si) as previously described (Lee et al., 2010). The schematic of promoter replacement was shown in Figure 4. First, a 1.2 kb geneticin resistance gene (G418) and a 0.8 kb Pzear fragment were amplified from plasmid pNEO and genomic DNA of PH-1 respectively and fused the two fragments to obtain the G418-Pzear fragment by double joint polymerase chain reaction (DJ-PCR) as previously described (Yu et al., 2004; Ren et al., 2014). Second, a 1.0 kb upstream region of *Fg*Stu2 original promoter (Pstu2) and a 1.0 kb *Fg*Stu2 coding sequence were amplified from genomic DNA of PH-1. Finally, the three fragments (upstream region of Pstu2, G418-Pzear and 1.0 kb *Fg*Stu2 coding sequence) were fused by DJ-PCR to obtain the Pstu2 substitution vector which was used for protoplast transformation. To induce Pzear replacement, 30 μM zearalenone (ZEA, Sigma Aldrich, St. Louis, MO, United States) was added to the medium during the regeneration (Lee et al., 2010).

### RNA Extraction and Quantitative Real-Time PCR

To test the expression level of *Fg*Stu2 gene, the mycelium was harvested after cultivation for 36 h in YEPD medium. This time is sufficient for gaining enough mycelium and distinguishing the expression level of *Fg*Stu2 gene in wild-type strain and *Fg*Stu2-Si mutants. To test the expression level of *Fg*Tri5 and *Fg*Tri6 genes, the mycelium was harvested after three-days cultivation in TBI medium at 28°C in the dark (Gardiner et al., 2009). The reason why we chose three days is that the level of DON increases obviously between 48 and 72 h and then increase slowly after 72 h and reaches the stationary phase (Tang et al., 2018). Total RNA extraction was follow the protocol of Total RNA Extraction Kit (Tiangen, China, DP419). First cDNA was synthesized by HiScript^®^ II Reverse Transcriptase (Vazyme, Nanjing, China, R223-01). The ChamQTM SYBR^®^ qPCR Master Mix (Vazyme, Nanjing, China, Q311-02/03) was used for Quantitative real-time PCR (qRT-PCR), which was conducted at CFX Connect Real-Time System (Bio-Rad) with the following procedure: 95°C 30 s; following 40 cycles of 95°C 10 s and 60°C 30 s; and a melt curve step of 95°C for 15 s and 60°C for 60 s, followed by 71 cycles of gradual increase in temperature from 60 to 95°C for 13 s/cycle. The primers used for qRT-PCR analysis are listed in Supplementary Table 1. The endogenous housekeeping gene *Fg*actin was used for normalization.

### Morphology Observation

For photograph the colonial morphology, 5-mm mycelial plugs from the margin of a 3-day-old colony (on PDA plates) of wild-type strain PH-1 and *FgStu2* silent mutant were transferred on PDA, MM, and CM plates with or without 30 mM ZEA and keep it at 25°C for three days. After that, the colonial morphology was photographed. For hyphal morphology, wild-type strain PH-1 and *Fg*Stu2 silent mutants were inoculated on PDA plates with or without 30 μM ZEA for 24 h. After that, the margin of the small colony was photographed using an Olympus IX-71 inverted microscope.

### Fluorescence Observation of *Fg*β1-Tubulin, *Fg*β2-Tubulin, and *Fg*Stu2-mRFP

To observe the localizations of *Fg*Stu2 and *Fg*β1-tubulin, *Fg*β2-tubulin, the fresh spores of *Fg*β2EGFP-Stu2mRFP were inoculated into 20 ml YEPD medium with or without 30 μM ZEA to germinate at 25°C for 10-12 h and then pipette 10 μl medium onto the slide for micro examination. This experiment was conducted by a LEICA TCS SP8 confocal laser-scanning microscope (LEICA).

### Growth Rate and MBC Sensitivity Tests

The growth rate was tested as previously described (Zhu et al., 2018). In brief, 5 mm mycelial plugs taken from the margin of a 3-day-old colony of wild-type strain PH1 and *Fg*Stu2 silent mutants were transferred into PDA plates with or without 30 μM ZEA and incubated at 25°C. After three days cultivation, the colony diameters of wild-type strain and *Fg*Stu2-Si mutants were significantly different. Then the diameters of each strain were measured to calculate the mean growth rates. This experiment was conducted three independent times.

Radial growth was used to test the sensitivity of the wild-type strain PH-1 and *Fg*Stu2 mutants to MBC as previously described (Zhu et al., 2018). Colony diameter was measured after 3 days for PH1; after 7 days for *Fg*Stu2-Si mutants. The MBC was added to PDA plates at 0.2, 0.4, 0.6, 0.8, 1.0, and 1.2 μg/ml for PH-1 and *Fg*Stu2-Si mutant; at 5, 10, 15, 20, and 25 μg/ml for *Fg*β2F167Y and F167Y-*Fg*Stu2-Si mutant.

### Assessment of Asexual Reproduction

In order to assess the ability of asexual reproduction of *Fg*Stu2-Si mutants, we observed the conidial morphology and tested the number of conidial production of *Fg*Stu2-Si mutant as previously described (Zhu et al., 2018). For observe the septa and nuclei, fresh spores were stained with calcofluor white (CFW) and 4′,6-diamidino-2-phenylindole (DAPI) and examined with a LEICA TCS SP8 confocal laser-scanning microscope (LEICA). The numbers of conidia produced by the wild-type strain PH-1 and *Fg*Stu2-Si mutants were calculated using hemocytometer as previously described (Zheng et al., 2014). This experiment was conducted three independent times.

### Assessment of Sexual Reproduction

Sexual reproduction test was performed as previous described (Bui et al., 2016). Briefly, inoculate fresh mycelia of each stain to carrot medium plates with or without 30 μM ZEA and keep it at 25°C incubator until the plates are full of mycelia. Then clean out the mycelia and add 2.5% Tween 60 solution to the plates. Keep the plates into 25°C incubator with near-UV light for about one or two weeks until the black perithecia are appeared and then photograph the plates by stereomicroscope. This experiment was conducted three independent times.

### Pathogenicity Assay

In order to compare the pathogenicity of the wild-type strain PH-1 and *Fg*Stu2-Si mutants we preformed wheat coleoptile infection assay as previous described (Zhang et al., 2012). In brief, Huaimai 33 wheat seeds were placed in culture dishes and placed it into a 25°C chamber with light. After 3 days, remove the 2-3 mm tip of all coleoptiles and the wound was inoculated with 2.5 μl (1 × 10^6^ spores/ml) fresh conidia, which was collected from MBL medium with or without 30 μM ZEA. Coleoptiles inoculated with MBL medium with or without 30 μM ZEA were used as control. After 7 to 10 days cultivation, the infected coleoptiles were photographed and the length of lesion was measured. At least 20 wheat seedlings were measured for each strain. This experiment was conducted three independent times.

### Assessment of DON Reproduction

Deoxynivalenol production of *Fg*Stu2-Si mutants were determined by enzyme linked immunosorbent assay (ELISA) followed the instruction of DON detection kit (Weisai Technology, Jiangsu, China). Briefly, two fresh 5 mm mycelial plugs taken from the margin of a 3-day-old colony of wild-type strain PH-1 and *Fg*Stu2-Si mutants were transferred into 30 ml TBI medium with or without 30 μM ZEA and cultured at 28°C, 175 rpm in the dark (3 repeats were set for each strain). After 7 days cultivation, the medium and mycelium was collected. The medium was used for DON determination, the mycelium was dried in the dryer for 48h. After that the dry weight of the mycelium was used for calculating the DON production (μg DON/g mycelium). The DON production of wild-type strain PH-1 was used as reference to generate relative DON production of *Fg*Stu2-Si mutants. The experiment was repeated three independent times.

For visualize the localization *Fg*Tri1p (also known as “toxisomes”) in FgStu2-Si mutant and wild-type strain PH-1, we cloned *Fg*Tri1 gene into PDL2 plasmid as previous described (Liu et al., 2017). The recombinant plasmid was identified by PCR, and sequencing. Then we transformed the recombinant plasmid into protoplasm of *Fg*Stu2-Si mutant and wild-type strain PH-1 to generate *Fg*Stu2-Si-Tri1-EGFP and PH-1-Tri1-EGFP strains. The transformants were identified by PCR, sequencing and fluorescence microscope. The identified *Fg*Stu2-Si-Tri1-EGFP and PH-1-Tri1-EGFP strains were inoculated into TBI medium and incubated for 2 days at 28°C, 175 rpm in the dark, the fresh mycelium were used for fluorescence observation. This experiment was conducted by a LEICA TCS SP8 confocal laser-scanning microscope (LEICA). This experiment was repeated three independent times.

## Results

### Identification and Amino Acid Sequence Analysis of *Fg*Stu2 Gene

The protein blast results of NCBI and Ensembl Fungi website suggested that the FGSG_10528 (gene bank ID: 23557431) is the Dis1/Stu2/XMAP215 family homolog gene in *F. graminearum*. The *Fg*Stu2 gene is predicted to encode an 888 amino acids protein (*Fg*Stu2p) which is similar with the lengths of *S. cerevisiae* Stu2 and *S. pombe* Dis1 (Figure 1A). Sequence analysis showed that FgStu2p contains two TOG domains at the amino terminal, TOG1 domain (Position 1 to 233) and TOG2 domain (position 273 to 505) (Figure 1A). The *Fg*Stu2-TOG1 domain shares only 32% identity with that of *S. cerevisiae* Stu2 (*Sc*Stu2) and *S. pombe* Dis1 (*Sp*Dis1). The *Fg*Stu2-TOG2 domain shares only 26 and 33% identity with that of *S. cerevisiae* Stu2 and *S. pombe* Dis1. However, the TOG1 domain of *Fg*Stu2p share 63 and 71% identify with that of *A. nidulans* alpA and Dis1/Stu2/XMAP215 homolog of *N. crassa* respectively (Figure 1B). The TOG2 domain of *Fg*Stu2 share 68 and 74% identity with that of *A. nidulans* alpA and Dis1/Stu2/Xmap215 homolog of *N. crassa* respectively, which are two folds higher than that of *S. cerevisiae* Stu2 and *S. pombe* Dis1 (Figure 1C). Moreover, The carboxyl terminal part of *Fg*Stu2p (position 506 to 877) shares 46 and 52% identity with that of *A. nidulans* alpA (position 510 to 891) and *N. crassa* stu2 homolog (position 509 to 941) which are nearly three times higher than the 18% identity with that of *S. cerevisiae* Stu2 (position 567 to 888) and *S. pombe* Dis1 (position 539 to 882) (Supplementary Figure 2).

### *Fg*Stu2p Is a Microtubule- and Kinetochore-Associated Protein

The *Fg*β-tubulin-GFP and RFP-*Fg*Stu2 double labeling experiment indicated that most of the *Fg*Stu2p was localized at cytoplasm and some of them were co-localized with free tubulin heterodimers which were not polymerized into microtubules. Another part of *Fg*Stu2p was co-localized with microtubules which contain *Fg*β1-tubulin or *Fg*β2-tubulin (Figure 2A). However, we could not identify the specific location of *Fg*Stu2p in the microtubule such as the microtubule plus end. The result of co-immunoprecipitation (Co-IP) assay demonstrated that the *Fg*Stu2p binds to tubulin heterodimers which contain *Fg*β1-tubulin and *Fg*β2-tubulin (Figure 2B). In addition, our Co-IP assay also suggested that *Fg*Stu2p binds to *Fg*γ-tubulin and *Fg*Ndc80p, component of kinetochore, which was previously reported in fission yeast (Figure 3; Hsu and Toda, 2011).

### Construction of *Fg*Stu2 Promoter Replacement Mutant in *F. graminearum*

After attempted for several times, we failed to obtain *Fg*Stu2 gene deletion mutant by homologous recombination method (data not shown). We therefore used promoter replacement strategy to study the genetic function of *Fg*Stu2 as previously described (Figure 4A). The transformants were identified by PCR and southern blot. The results indicate that the promoter of *Fg*Stu2 gene has been replaced by G418 resistance gene and zear promoter (Figures 4B,C). The expression level of *Fg*Stu2 promoter replacement mutants (*Fg*Stu2-Si mutants) was significantly decreased compared with those of wild-type strain PH-1 and *Fg*β2F167Y strain. Addition of 30 μM ZEA partially restored the *Fg*Stu2 gene expression level (Figure 4D). These results indicate that we have successfully obtained the *Fg*Stu2-Si mutants.

### *Fg*Stu2 Gene Is Involved in Hyphal Morphology, Vegetative Growth, and Microtubule Polymerization of *F. graminearum*

**FIGURE 5:**
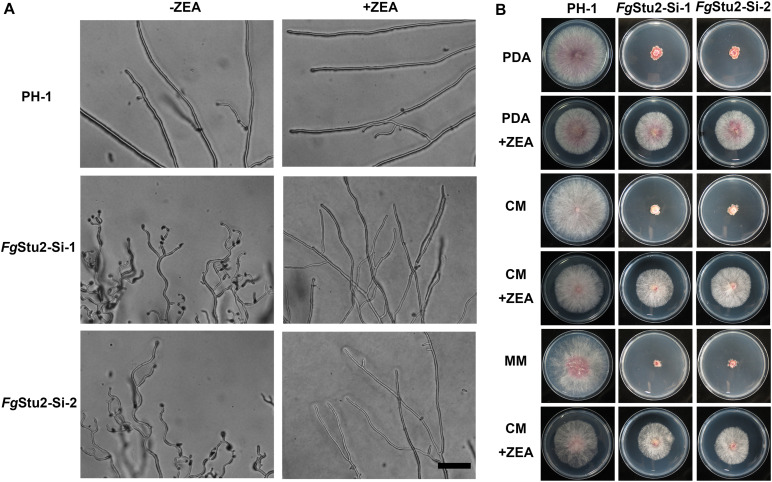
Roles of *Fg*Stu2 gene in hyphal and colonial morphology. **(A)** Hyphal morphology was photographed after strains were incubated on potato dextrose agar (PDA) plants with or without 30 μM zearalenone (ZEA) for 24 h at 25°C. Bar = 50 μm. **(B)** Colony morphology was photographed after strains were inoculated on potato dextrose agar (PDA), complete medium (CM), and minimal medium (MM) plants with or without 30 μM ZEA for 3 days at 25°C.

**FIGURE 6:**
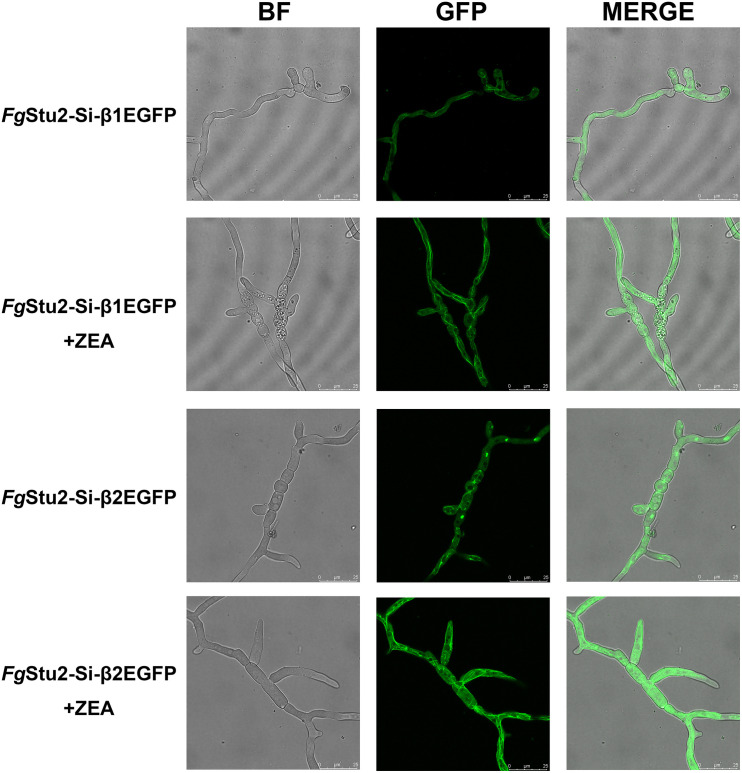
Roles of *Fg*Stu2 gene in microtubule polymerization. Fresh spores (10^5^) of *Fg*Stu2-Si-β1EGFP and *Fg*Stu2-Si-β2EGFP were first incubated into 20 ml of yeast extract-peptone-dextrose medium (YEPD) with or without 30 μM zearalenone (ZEA) and then put it on an orbital shaker (175 rpm) at 25°C to germinate for 10 h and then spotted 5 μl of culture onto the slides for confocal micro examination. Bar = 25 μm.

The *Fg*Stu2-Si mutants produced twisted hyphae compared with that of the wild-type strain PH-1. Addition of 30 μM ZEA restored the hyphal morphology (Figure 5A). Moreover, the *Fg*Stu2-Si mutants grew significantly slower than the wild-type strain PH-1 on PDA, MM, and CM plates. Addition of 30 μM ZEA partially restored the growth rate (Figure 5B and Table 2). Besides, microtubule network was sparse in *Fg*Stu2-Si mutants, which was recovered by addition of 30 μM ZEA (Figure 6).

**TABLE 2:** Hyphal growth rate and sensitivity of wild-type strain PH1, *Fg*Stu2 promoter replacement mutants to MBC on PDA with or without 30 μM zearalenone (ZEA).

Strains and mutants	Growth Rates (cm/day)^a^		EC_50_ (μg/ml)^b^	
		
	−ZEA	+ZEA	−ZEA	+ZEA
PH1	2.62 ± 0.10a	2.02 ± 0.18b	0.4954 ± 0.0198b	0.6780 ± 0.0177a
*Fg*Stu2-Si-1	0.51 ± 0.09d	1.47 ± 0.22c	0.4184 ± 0.0435bc	0.6150 ± 0.0513a
*Fg*Stu2-Si-2	0.53 ± 0.11d	1.51 ± 0.12c	0.4090 ± 0.0352c	0.6181 ± 0.0525a

*^*a*^Growth rate was measured after incubation of three replicates for 3 days. ^*b*^MBC concentration that resulted in 50% mycelial growth inhibition. Values are means and standard deviations. Means in the table followed by the same letter are not significantly different (*P* < 0.05).*

### FgStu2-Si Mutants Had Slightly Decreased MBC Sensitivity

We tested MBC sensitivity of *Fg*Stu2 promoter replacement mutants. The effect concentration against 50% mycelia growth (EC_50_ value) of *Fg*Stu2-Si mutants was slightly decreased compared with those of wild-type strain PH-1 (Table 2). However, the *Fg*Stu2-Si mutants cannot grow on PDA containing 0.8 μg/ml MBC. When the PDA plates was added 30 μM ZEA, the EC_50_ values *Fg*Stu2-Si mutants were restored and even a bit higher than that of wild-type strain PH-1 (Supplementary Figure 6A). The same phenomenon was observed in *Fg*Stu2 promoter replacement mutant generated from *Fg*β2F167Y (*Fg*β2F167Y-Stu2-Si), an MBC resistant strain reported previously. The EC_50_ value of *Fg*β2F167Y-Stu2-Si (4.28 ± 0.32 μg/ml) was decreased about 30% compared with that of parental strain *Fg*β2F167Y (6.48 ± 0.27 μg/ml) (Table 3). Moreover, the *Fg*β2F167Y-*Fg*Stu2-Si cannot grow on PDA plates containing 20 μg/ml MBC (Supplementary Figure 6B).

**TABLE 3:** Hyphal growth rate and sensitivity of *Fg*β2F167Y and *Fg*β2F167Y-Stu2-Simutant to MBC^∗^ on PDA with or without 30 μM zearalenone (ZEA).

Strains and mutants	Growth Rates (cm/day)^a^		EC_50_ (μ g/ml)^b^	
		
	−ZEA	+ZEA	−ZEA	+ZEA
*Fg*β2F167Y	2.61 ± 0.17a	2.01 ± 0.19b	6.4808 ± 0.2740b	7.9354 ± 0.3017a
*Fg*β2F167Y-Stu2-Si	0.54 ± 0.12d	1.53 ± 0.11c	4.2803 ± 0.3255d	5.546 ± 0.4215c

*^*a*^Growth rate was measured after incubation of three replicates for 3 days. ^*b*^MBC concentration that resulted in 50% mycelial growth inhibition. Values are means and standard deviations. Means in the table followed by the same letter are not significantly different (*P* < 0.05).*

### The *Fg*Stu2 Gene Is Involved in Sexual and Asexual Reproduction of *F. graminearum*

**FIGURE 7:**
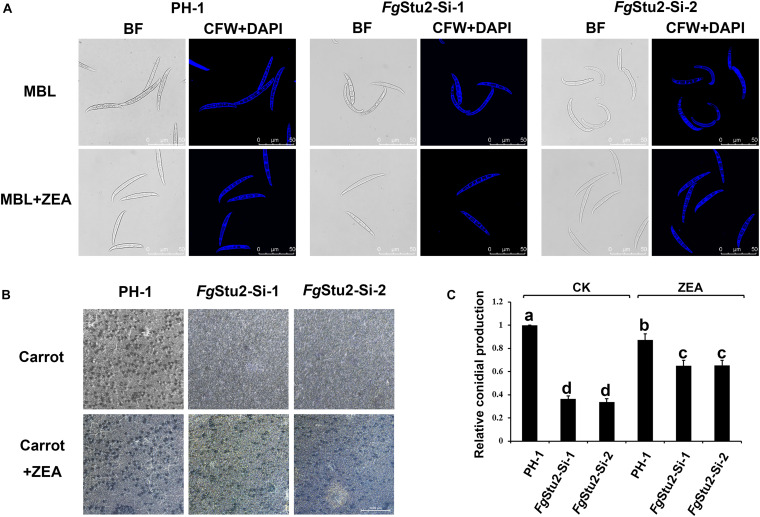
Roles of *Fg*Stu2 gene in sexual and asexual reproduction. **(A)** Conidial morphology of wild-type strain PH-1, *Fg*Stu2-Si mutants. The conidia of all strains were harvested after 3-days cultivation in mung bean liquid (MBL) medium with or without 30 μM zearalenone (ZEA) at 25°C. Fresh conidia were first stained with calcofluor white (CFW) and 4’,6-diamidino-2-phenylindole (DAPI) and then photographed using a confocal laser-scanning microscope. Bar = 50 μm. **(B)** Ability of perithecia production of wild-type strain PH-1, *Fg*Stu2-Si-1, *Fg*Stu2-Si-2. Inoculate fresh mycelia of each stain to carrot medium plates with or without 30 M m ZEA and keep it at 25°C incubator until the plates are full of mycelia. Then clean out the mycelia and add 2.5% Tween 60 solution to the plates. Keep the plates into 25°C incubator with near-UV light for about one or two weeks until the black perithecia were appeared and then photograph the plates by stereomicroscope. Bar = 1000 μm. **(C)** Numbers of conidial production of wild-type strain PH-1, *Fg*Stu2-Si-1, *Fg*Stu2-Si-2. Conidial production was measured by hemacytometer after three days incubation in mung bean liquid (MBL) medium with or without 30 μM ZEA. Means of the column with the same letter are not significantly different (*P* < 0.05).

The *Fg*Stu2 gene promoter replacement mutant showed curved conidia and decreased number of conidial production compared with those of the wild-type strain PH-1. While the conidial morphology and number of conidial production were restored after addition of 30 μM ZEA (Figures 7A,C). Moreover, the *Fg*Stu2-Si mutants lost ability to produce perithecia. Addition of 30 μM ZEA restored the ability of perithecia production (Figure 7B).

### The *Fg*Stu2 Gene Is Involved in Virulence of *F. graminearum*

**FIGURE 8:**
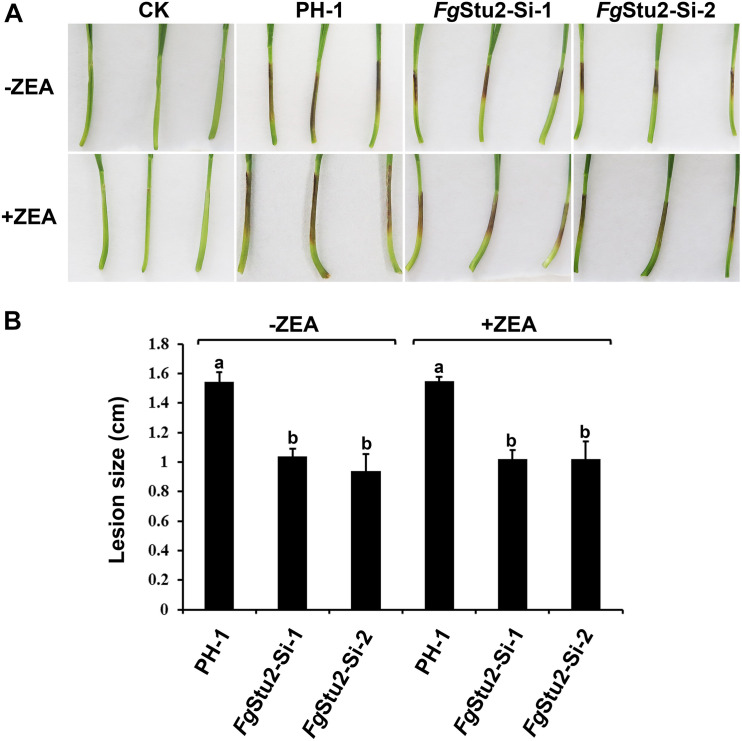
Roles of *Fg*Stu2 gene in pathogenicity. **(A)** Infected wheat coleoptiles were photographed at 7 days after inoculation. The small solid arrows indicate the inoculation sites and the hollow arrow indicates the lesion development direction. **(B)** Lengths of brown lesions of wheat coleoptiles. Values are means ± standard deviation of three repeated experiments. Means of the column with the same letter are not significantly different (*P* < 0.05).

We performed the coleoptile infection assay to assess the rule of *Fg*Stu2 gene in pathogenicity of *F. graminearum*. The lesion length of coleoptiles inoculated with conidia of *Fg*Stu2-Si mutants was decreased about 35% compared with those of the wild-type strain PH-1 (Figure 8A). However, the conidia of *Fg*Stu2-Si mutants collected from MBL medium containing 30 μM ZEA did not cause the same size of lesion (Figures 8A,B).

### The *Fg*Stu2 Gene Is Involved in DON Production of *F. graminearum*

**FIGURE 9:**
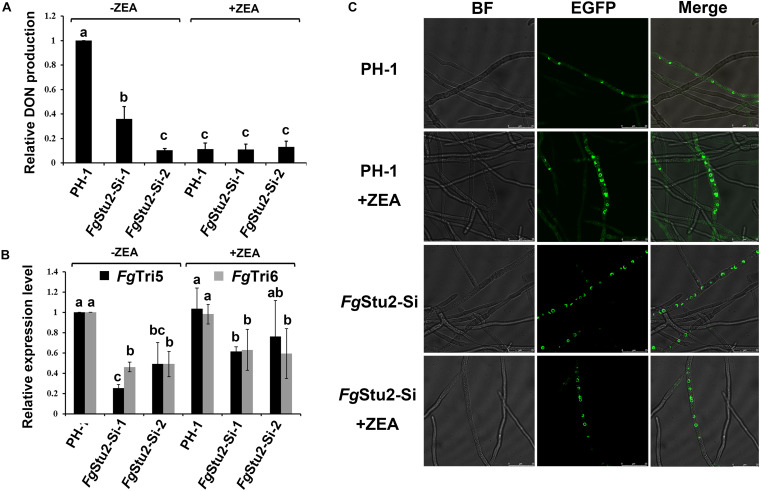
Roles of *Fg*Stu2 gene in DON production. **(A)** Relative DON production of *Fg*Stu2-Si mutants. Wild-type strain PH-1 and *Fg*Stu2-Si mutants were inoculated into TBI medium with or without 30 μM zearalenone (ZEA) and cultivate for 7 days at 28 °C in the dark. DON production was tested using enzyme linked immunosorbent assay (ELISA). Means come from three independent assays. **(B)** Relative expression levels of *Fg*Tri5 and *Fg*Tri6 genes in *Fg*Stu2-Si mutants. Wild-type strain PH-1 was used as control. Wild-type strain PH-1 and *Fg*Stu2-Si mutants were inoculated into TBI medium with or without 30 μM ZEA and cultivate for 3 days at 28 °C in the dark. The fresh mycelium was used for total RNA extraction. Means come from three independent assays. **(C)** *Fg*Tri1-EGFP localization in wild-type strain PH-1 and *Fg*Stu2-Si mutants. PH-1-Tri1-EGFP and *Fg*Stu2-Si-Tri1EGFP strains were inoculated into TBI medium with or without 30 μM ZEA and cultivate for 2 days at 28 °C in the dark. The fresh mycelium was observed using a Leica SP8 laser confocal microscope. Bar = 25 μm. This experiment was repeated three times.

The DON production was significantly decreased in *Fg*Stu2-Si mutants compared with that of wild-type strain PH-1 (Figure 9A). However, addition of ZEA didn’t restore the DON production of *Fg*Stu2-Si mutants and decreased the DON production of PH-1. Moreover, expression levels of trichothecene biosynthesis pathway genes (Tri gene) including *Fg*Tri5 and *Fg*Tri6 were significantly decreased in *Fg*Stu2-Si mutants (Figure 9B). The localization of Tri1-EGFP, also known as “toxisome,” in *Fg*Stu2-Si mutant was similar with that of wild-type strain PH-1 (Figure 9C).

## Discussion

In this study, we identified the *Fg*Stu2 gene (FGSG_10528) is the Dis1/Stu2/XMAP215 microtubule associated protein family homolog of *F. graminearum*. The amino acid sequences of two *Fg*Stu2-TOG domains share 63 and 74% identity with that of *A. nidulans* and *N. crassa*, which is much higher than the 26 and 33% identity with *Sc*Stu2 and *Sp*Dis1 (Figure 1B). The similar result was also reflected in the sequence alignment of carboxyl terminal area. These results indicate that the *Fg*Stu2 is more homologous to Dis1/Stu2/XMAP215 homologs in filamentous fungi than yeast (Supplementary Figure 1). Moreover, we tried to substitute *FgStu2* in wild-type strain PH-1 *with Sc*Stu2. However, after several attempts, we didn’t acquire the substitution mutant which means *Sc*Stu2 might not exert the function of *Fg*Stu2. This phenomenon may due to the different tubulin structures and sequences of *F. graminearum* and yeast.

Previous studies demonstrated that Dis1/Stu2/XMAP215 homologs binds to tubulin and microtubule (Al-Bassam and Chang, 2011; Widlund et al., 2011; Thawani et al., 2018). We therefore examined whether *Fg*Stu2p interacts with *Fg*β-tubulin physically. The co-localization experiment and co-immunoprecipitation demonstrated that *Fg*Stu2p binds to *Fg*β1-tubulin and *Fg*β2-tubulin and partly localizes to microtubule in hyphae of *F. graminearum* (Figures 2A,B). However, due to the microtubule network in *F. graminearum* is more complicated than that of yeast, we couldn’t confirm the microtubule plus end tracking property of *Fg*Stu2 under our experiment condition. At least, we demonstrated the microtubule associated property of *Fg*Stu2p in *F. graminearum*.

In order to investigate the functional role of *Fg*Stu2 gene in *F. graminearum*, we firstly tried to disrupt *Fg*Stu2 gene by gene replacement strategy. However, we cannot acquire correct transformants after several attempts (data not shown), which means *Fg*Stu2 gene might be essential for *F. graminearum*. This phenomenon is consistent with the result of Stu2 disruption experiment in *S. cerevisiae* (Wang and Huffaker, 1997; Al-Bassam et al., 2006). However, deletion of XMAP215 homologs in fission yeast and *A. nidulans* is viable (Enke et al., 2007; Hsu and Toda, 2011). These results indicate that functional importance of XMAP215 homologs in different fungal species is divergent. Another possibility is that some species may contain more than one XMAP215 homolog. For example, fission yeast contains two XMAP215 homologs: Dis1 and Alp14 genes, delete any one of them would not be lethal (Hsu and Toda, 2011; Flor-Parra et al., 2018). Therefore, we preformed promoter replacement strategy to study the *Fg*Stu2 gene. The promoter replacement mutant *Fg*Stu2-Si exhibited drastically decreased growth rate compared with that of wild-type strain PH-1. According to a previous review, loss functions of the XMAP215 homologs leads to decreased growth rates in many species (Al-Bassam and Chang, 2011). Our results are identical with these studies which indicate that *FgStu2* gene is essential for the viability of *F. graminearum.*

Besides exhibited decreased growth rate, the *Fg*Stu2-Si also produced twisted hyphae and curved conidia (Figures 5A, 7A). According to previous studies, benomyl (a kind of benzimidazole fungicide, which can destabilize microtubules) treatment can destabilize microtubule, affect hyphal tip growth of *A. nidulans*, and develop twisted hyphae (Horio and Oakley, 2005); disrupt the *Fg*EB1 (Ending binding one protein homolog in *F. graminearum*, another kind of conserved microtubule-associated protein) gene resulted in less organized microtubule and therefore partly caused the twisted hyphae and curved conidia (Liu et al., 2017); the *Fg*α1-tubulin and *Fg*β2-tubulin gene deletion mutants displayed twisted hyphae and abnormal conidia (Hu et al., 2015; Zhu et al., 2018). These results suggest that microtubule plays a vital role in morphology of filamentous fungi. Moreover, the alpA gene (Dis1/Stu2/XMAP215 homolog in *A. nidulans*) deletion mutant exhibited reduced microtubule polymerization and twisted hyphae (Enke et al., 2007). We therefore wonder whether the aberrant hyphae and conidia of *Fg*Stu2-Si mutant are caused by abnormal microtubule polymerization. As our expected, The *Fg*Stu2-Si mutant exhibited less organized microtubule (Figure 6). This result is consistent with other studies about XMAP215 homologs in other species (Al-Bassam and Chang, 2011; Thawani et al., 2018). We therefore conclude that *Fg*Stu2 regulates the morphology of *F. graminearum* via microtubule associated function. Although the microtubule polymerization was decreased in *Fg*Stu2-Si mutants, the *Fg*Stu2-Si mutant didn’t show highly increased sensitivity to microtubule destabilizing fungicide MBC (Tables 2, 3 and Supplementary Figure 6). This result indicates that the existed microtubule in *Fg*Stu2-Si did not exhibit highly increased sensitivity to microtubule-destabilizing effect of MBC. Previous study showed that AlpA deletion mutant of *A. nidulans* exhibited increased sensitivity to benomyl (Enke et al., 2007). However, in this study, we didn’t acquire the deletion mutant of *Fg*Stu2. The *Fg*Stu2 expression was not completely eliminated in *Fg*Stu2-Si mutants (Figure 4D), which may still contribute to stabilize microtubule of *Fg*Stu2-Si mutant. Another possibility is that the microtubule polymerization promoting activity of *Fg*Stu2p is too limited to resist the microtubule-depolymerizing activity of MBC. Although *Fg*Stu2-Si mutants are not hypersensitive to MBC, they was unable to grow on PDA plates containing 0.8 μg/ml MBC and *Fg*β2F167Y-Stu2-Si also showed increased sensitivity to MBC (Supplementary Figure 6). Therefore, *Fg*Stu2 plays a role, but not a major role, in regulation of MBC sensitivity of *F. graminearum*.

Besides the abnormal conidial morphology, the numbers of conidia produced by *Fg*Stu2-Si mutants were decreased compared with that of wild-type strain PH-1. Moreover, *Fg*Stu2-Si mutants did not produce perithecia. These results indicate that repression of *Fg*Stu2 gene affects both the sexual and asexual reproduction. Therefore, the cytokinesis of *F. graminearum* must be disturbed in *Fg*Stu2-Si mutants. According to previous studies, microtubule nucleation is vital to formation of spindle microtubules and the XMAP215 involves microtubule nucleation by interaction with γ-tubulin ring complex (Thawani et al., 2018); interaction between spindle microtubule and kinetochore is important for chromosome segregation during cytokinesis and the Stu2 regulates this interaction through interacting with Ndc80 complex of kinetochore (Miller et al., 2016); the Dis1 gene is required for proper meiotic chromosome disjunction in fission yeast (Rockmill and Fogel, 1988); In *Drosophila*, the msps gene (XMAP215 homolog in Drosophila) mutant displayed defect in chromosome segregation (Cullen and Ohkura, 2001); In human HELA cell line, deletion of XMAP215 homolog TOGp affects the integrity of centrosomes and spindle poles and delays mitosis (Cassimeris and Morabito, 2004). These studies indicate that loss functions of Dis1/Stu2/XMAP215 homologs in different species will disturb cytokinesis which is similar with our results. Moreover, we also demonstrated that the *Fg*Stu2 interacts with *Fg*γ-tubulin and presumed *Fg*Ndc80 which suggests that *Fg*Stu2 may involve the cytokinesis associated functions of *Fg*Ndc80 and *Fg*γ-tubulin just like XMAP215 homologs in other species.

Previous studies showed that deletion mutants of many genes in *F. graminearum*, such as *Fg*α1-tubulin, *Fg*β2-tubulin, and *Fg*TBCA (Tubulin folding cofactor A homolog in *F. graminearum*) etc., have dramatically decreased growth rates and are almost incapable of infecting host (Hu et al., 2015; Zhang et al., 2015; Zhu et al., 2018). Similar with these studies, the mean growth rates of *Fg*Stu2-Si mutants (0.5 cm/day) were decreased about 80% compared with that of wild-type strain PH-1 (2.6 cm/day) (Figure 5B and Table 2). However, the mean lengths of lesion caused by the *Fg*Stu2-Si mutants (1 cm) were decreased only about 35% compared with that of wild-type strain PH-1 (1.5 cm), which indicates that the *Fg*Stu2-Si mutants are still capable of infecting host (Figure 8). We considered the growth of *Fg*Stu2-Si mutants might be recovered during invasive growth in coleoptile. A previous study showed that the expression level of many genes of *F. graminearum* were upregulated during invasive growth *in planta*. Among them, the expression level of FGSG_04581 gene of PH-1 growing *in planta* has upregulated 6.79 times compared with that of PH-1 growing *in vitro* (Zhang et al., 2012). According to another study, the expression level of the FGSG_04581 gene is regulated by the zearalenone (Lee et al., 2010). Therefore, the zearalenone concentration in the hyphae maybe increased during infection. Interestingly, the expression of the *Fg*Stu2 gene in *Fg*Stu2-Si mutants is control by the same promoter of FGSG_04581 gene, which indicates that the expression of *Fg*Stu2 gene was likely to be restored by the increased zearalenone concentration and therefore restored the growth and pathogenicity. Nonetheless, we still observed the decreased pathogenicity of *Fg*Stu2-Si mutant. The dramatically decreased growth rate would be detrimental to the virulence of *F. graminearum.* Besides the growth rates, microtubules also play a crucial role in pathogenicity. Previous studies showed that, microtubule is important for transportation of secondary metabolites of plant pathogenic fungi (Conesa et al., 2001; Chanda et al., 2009). In addition, *Fg*EB1 deletion mutants showed less organized microtubule and decreased pathogenicity (Liu et al., 2017). In this study, microtubule polymerization was decreased in *Fg*Stu2-Si mutant, which may affect the transportation of virulence-associated factors such as effective protein and DON and therefore contributed to the decreased pathogenicity. However, spores of *Fg*Stu2-Si mutant collected from MBL medium containing zearalenone did not caused the same size of lesion compared with that of PH-1 which is similar with the *Fg*HSP90 knockdown mutant (Bui et al., 2016). This is likely due to the *Fg*Stu2p was not enough during invasion and therefore showed decreased pathogenicity. Future study should find another way to replace the method of addition of zearalenone.

The DON was wildly considered as an important virulence factor of *F. graminearum* (Chen et al., 2019). In this study, the DON production of *Fg*Stu2-Si mutants were significantly decreased which means the *Fg*Stu2 gene is involved the DON production (Figure 9A). Previous studies showed that the expression level of Tri genes were associated with the DON production (Chen et al., 2019). In this study, the expression level of *Fg*Tri5 and *Fg*Tri6 were decreased in *Fg*Stu2-Si mutants, which is consistent with previous studied. However, the *Fg*Tri1 localization, which is known as “toxisomes,” has not been affected in *Fg*Stu2-Si mutant (Menke et al., 2013), which is different from the *Fg*Tri1 localization of *Fg*β2-tubulin and *Fg*EB1 deletion mutants (Liu et al., 2017; Wang et al., 2019). This result indicates that different microtubule-associated proteins may play divergent roles in DON synthesis pathway, which needs further investigation. Nonetheless, the decreased microtubule polymerization *Fg*Stu2-Si mutant would disturb the transportation of DON, which is similar with *Fg*EB1 deletion mutants. It is strange that addition of zearalenone decreased the DON production of wild-type strain PH-1 and didn’t restore the DON production of *Fg*Stu2-Si mutant and PH-1. This phenomenon indicates that addition of zearalenone might result in a negative regulation of DON synthesis of *F. graminearum*.

In summary, this study revealed the key roles of *Fg*Stu2 gene in phenotypes, such as vegetative growth, morphology, sexual reproduction, asexual reproduction, pathogenicity and DON production of *F. graminearum*. We also demonstrated the microtubule-associated property of *Fg*Stu2, which is vital to the vegetative growth and morphology. Our results supplemented the functional roles of Dis1/Stu2/XMAP215 homologs in plant pathogenic fungi, which enriched the theory of Dis1/Stu2/XMAP215 family. Future studies should go deep into the role of *Fg*Stu2 and other microtubule-associated proteins in DON and ZEA production, which could be useful for developing new kinds of fungicide to control the mycotoxin contamination.

## Data Availability Statement

The original contributions presented in the study are included in the article/Supplementary Material, further inquiries can be directed to the corresponding author.

## Author Contributions

YYZ, YSZ, and MZ conceived and designed the experiments. NL, WR, YH, and XS guided the methods. YYZ and YSZ performed the experiments, analyzed the data, and prepared the figures and tables. YYZ and MZ wrote the manuscript. YSZ, WR, YH, YD, and MZ revised the manuscript. All authors have read and approved the final manuscript.

## Conflict of Interest

The authors declare that the research was conducted in the absence of any commercial or financial relationships that could be construed as a potential conflict of interest.

## Supplementary Material

Supplementary Figure 1 | The phylogenetic analysis of Dis1/Stu2/XMAP215 homologs. The phylogenetic tree was generated by MEGA 7.0 software. The whole sequences of Dis1/Stu2/XMAP215 homologs (*Aaspergillus nidulans* protein ID: XP_663125.1; *Fusarium graminearum* protein ID: XP_011319522.1; *Neurospora crassa* protein ID: XP_956946.3; *Saccharomyces cerevisiae* protein ID: NP_013146.1; and *Schizosaccharomyces pombe* protein ID: NP_587785.1) were acquired from the National Center for Biotechnology information website (NCBI, https://www.ncbi.nlm.nih.gov/).

Supplementary Figure 2 | Multiple Sequences alignment of C-terminal areas of Stu2 homologs. Multiple sequences (*Aaspergillus nidulans* protein ID: XP_663125.1; *Fusarium graminearum* protein ID: XP_011319522.1; *Neurospora crassa* protein ID: XP_956946.3; *Saccharomyces cerevisiae* protein ID: NP_013146.1; and *Schizosaccharomyces pombe* protein ID: NP_587785.1) alignment was performed using the online service (www.genome.jp/tools-bin/ clustalw). The alignment images were generated by the online service of ESPript 3.0 (espript.ibcp.fr/ESPript/cgi-bin/ESPript.cgi).

Supplementary Figure 3 | Sequences alignment of budding yeast Ndc80 and presumed *Fg*Ndc80. Amino acid sequences (budding yeast Ndc80 protein ID: NP_012122.3; *Fg*Ndc80 protein ID: XP_011328506.1) alignment was performed using the online service (www.genome.jp/tools-bin/clustalw). The alignment images were generated by the online service of ESPript 3.0 (espript.ibcp.fr/ESPript/cgi-bin/ESPript.cgi).

Supplementary Figure 4 | Construction of the *Fg*Stu2-3 × FLAG strain. **(A)** Schematic of vector construction. **(B)** Polymerase chain reaction (PCR) identification of *Fg*Stu2-3 × FLAG strains. a 1.8 kb fragment was amplified by primers P29/P30 indicates that 3 × FLAG-G418 fragment was successfully inserted at 3′-terminal of *Fg*Stu2 coding sequence.

Supplementary Figure 5 | Construction of the *Fg*γ-tubulin-EGFP and *Fg*Ndc80-EGFP strains. **(A)** Schematic of vector construction. **(B)** Polymerase chain reaction (PCR) identification of *Fg*γ-tubulin-EGFP and *Fg*Ndc80-EGFP strains. a 2.7 kb fragment was amplified by primers P35/P36 indicates that the EGFP-HPH fragment was successfully inserted at 3′-terminal of *Fg*γ-tubulin coding sequence. a 2.7 kb fragment was amplified by primers P41/P42 indicates that the EGFP-HPH fragment was successfully inserted at 3′-terminal of presumed *Fg*Ndc80 coding sequence.

Supplementary Figure 6 | MBC Sensitivity of wild-type strain PH-1, *Fg*β 2F167Y, and FgStu2-Si mutants. **(A)** MBC Sensitivity of wild-type strain PH-1 and *Fg*Stu2-Si-1 on PDA plates with or without 30 μM zearalenone (ZEA). **(B)** MBC Sensitivity of *Fg*β2F167Y and F167Y-Stu2-Si on PDA plates with or without 30 μM ZEA. Plugs were transferred from the margins of 3-day old PDA colonies to fresh PDA plants containing different concentrations of MBC. The timing for photography was based on the growth rates of each strain.

Supplementary Table 1 | Primers used in this study.
