# Supplementary figures and images for: The Dis1/Stu2/XMAP215 Family Gene FgStu2 Is Involved in Vegetative Growth, Morphology, Sexual and Asexual Reproduction, Pathogenicity and DON Production of Fusarium graminearum
Source: Front Microbiol. 2020 Nov 20;11:545015. doi: 10.3389/fmicb.2020.545015 (PMC7714731; doi:10.3389/fmicb.2020.545015)

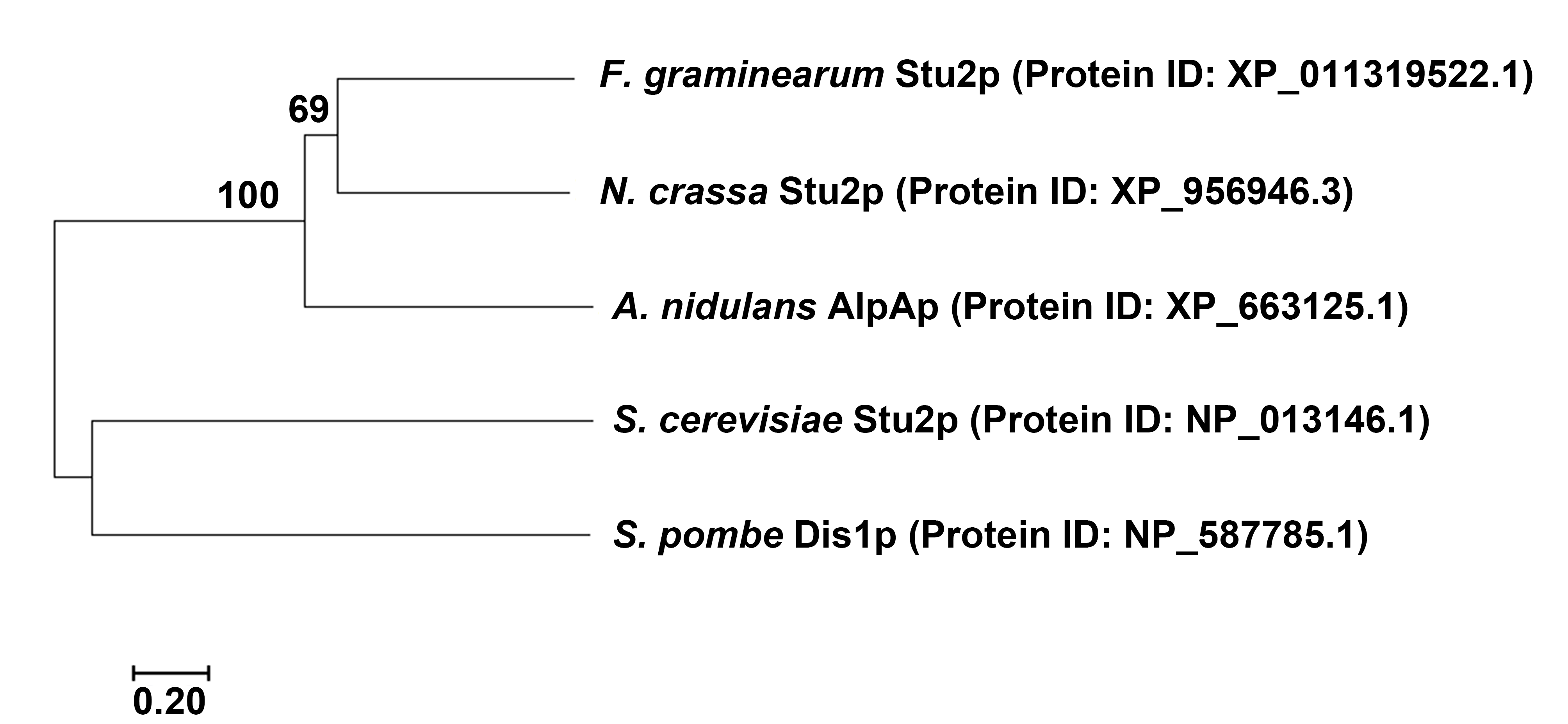

Supplement: Supplementary Figure 1 | — The phylogenetic analysis of Dis1/Stu2/XMAP215 homologs. The phylogenetic tree was generated by MEGA 7.0 software. The whole sequences of Dis1/Stu2/XMAP215 homologs (Aaspergillus nidulans protein ID: XP_663125.1; Fusarium graminearum protein ID: XP_011319522.1; Neurospora crassa protein ID: XP_956946.3; Saccharomyces cerevisiae protein ID: NP_013146.1; and Schizosaccharomyces pombe protein ID: NP_587785.1) were acquired from the National Center for Biotechnology information website (NCBI, https://www.ncbi.nlm.nih.gov/). [file Image_1.TIF]

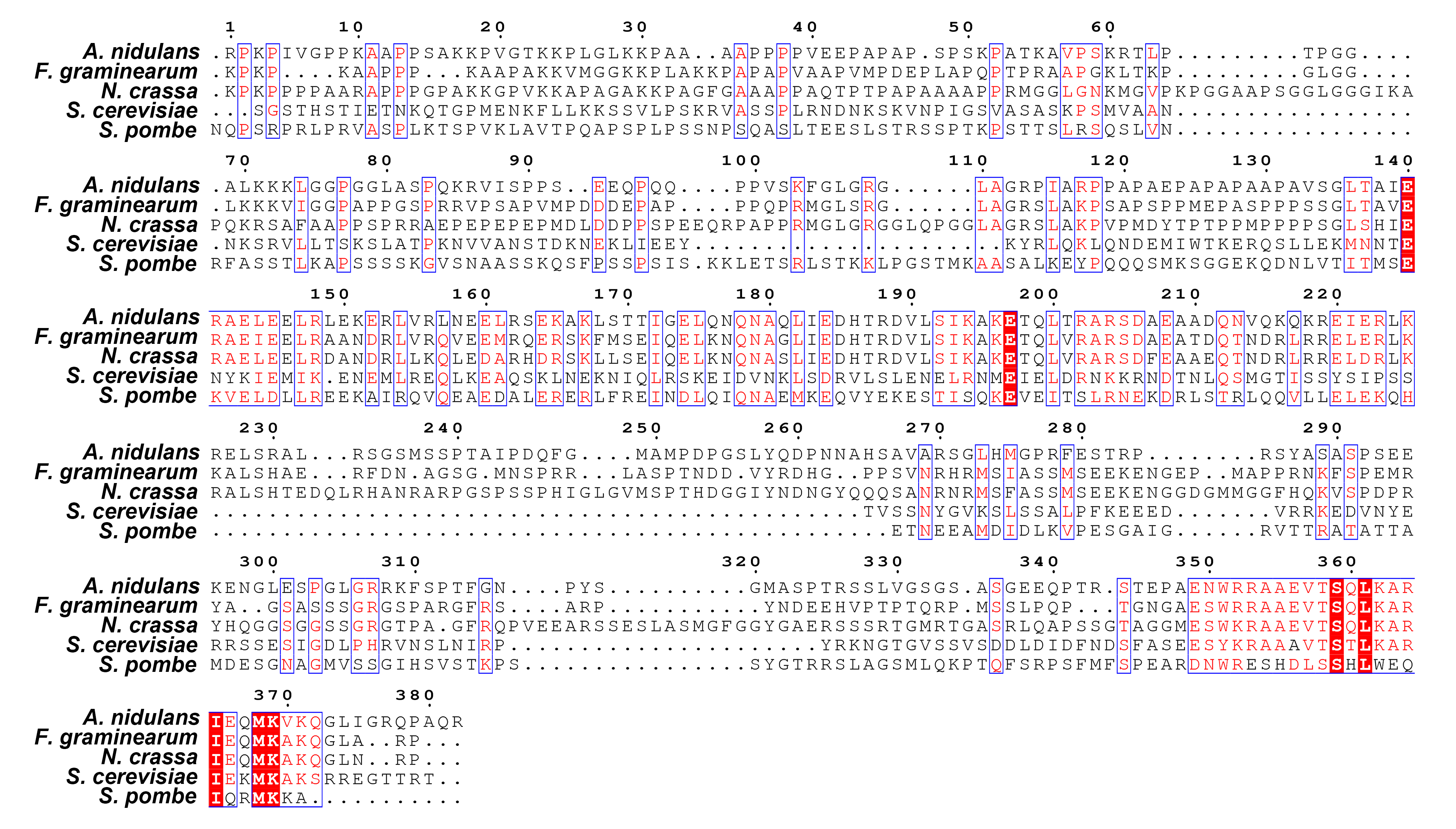

Supplement: Supplementary Figure 2 | — Multiple Sequences alignment of C-terminal areas of Stu2 homologs. Multiple sequences (Aaspergillus nidulans protein ID: XP_663125.1; Fusarium graminearum protein ID: XP_011319522.1; Neurospora crassa protein ID: XP_956946.3; Saccharomyces cerevisiae protein ID: NP_013146.1; and Schizosaccharomyces pombe protein ID: NP_587785.1) alignment was performed using the online service (www.genome.jp/tools-bin/ clustalw). The alignment images were generated by the online service of ESPript 3.0 (espript.ibcp.fr/ESPript/cgi-bin/ESPript.cgi). [file Image_2.TIF]

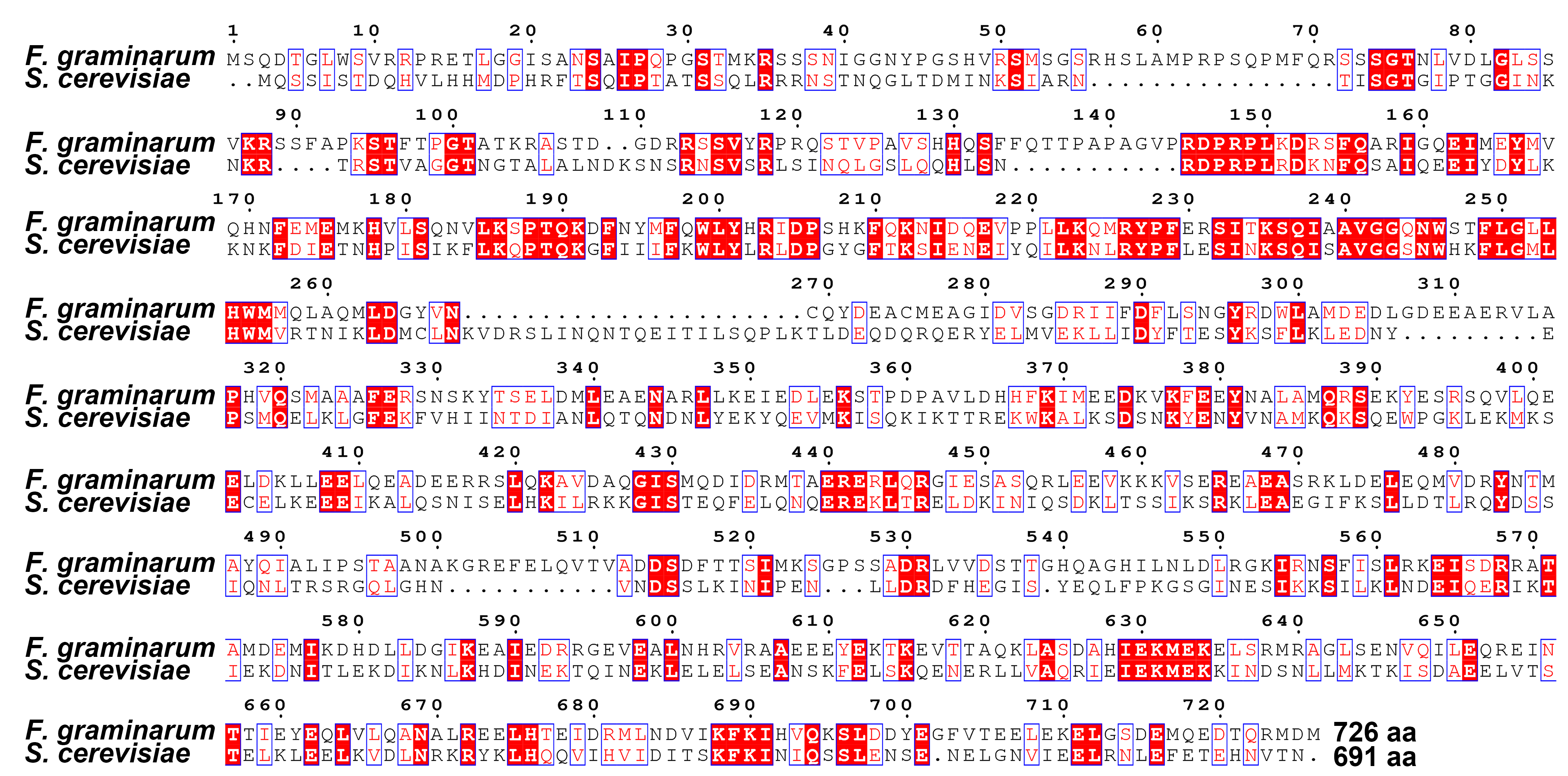

Supplement: Supplementary Figure 3 | — Sequences alignment of budding yeast Ndc80 and presumed FgNdc80. Amino acid sequences (budding yeast Ndc80 protein ID: NP_012122.3; FgNdc80 protein ID: XP_011328506.1) alignment was performed using the online service (www.genome.jp/tools-bin/clustalw). The alignment images were generated by the online service of ESPript 3.0 (espript.ibcp.fr/ESPript/cgi-bin/ESPript.cgi). [file Image_3.TIF]

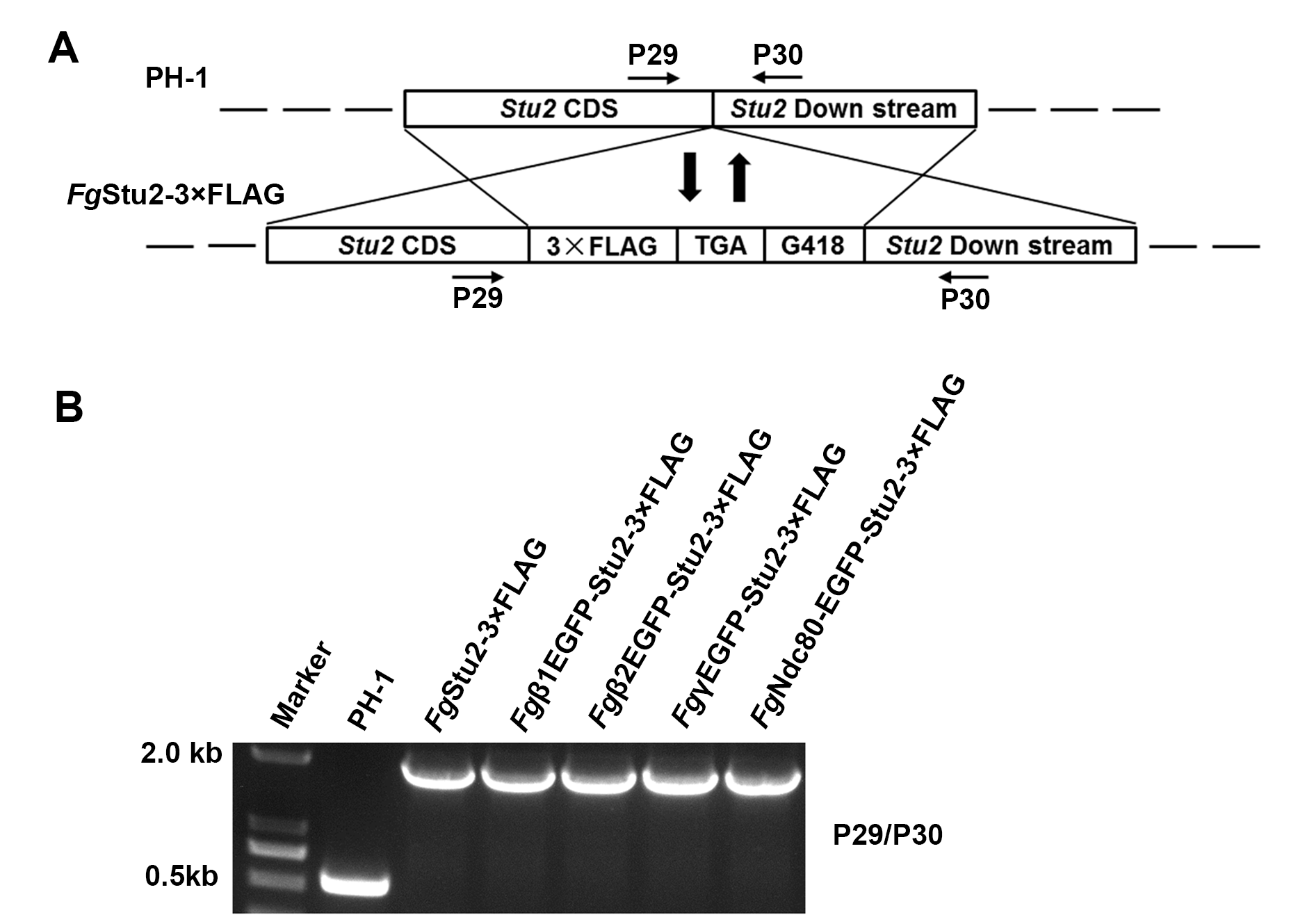

Supplement: Supplementary Figure 4 | — Construction of the FgStu2-3 × FLAG strain. (A) Schematic of vector construction. (B) Polymerase chain reaction (PCR) identification of FgStu2-3 × FLAG strains. a 1.8 kb fragment was amplified by primers P29/P30 indicates that 3 × FLAG-G418 fragment was successfully inserted at 3′-terminal of FgStu2 coding sequence. [file Image_4.TIF]

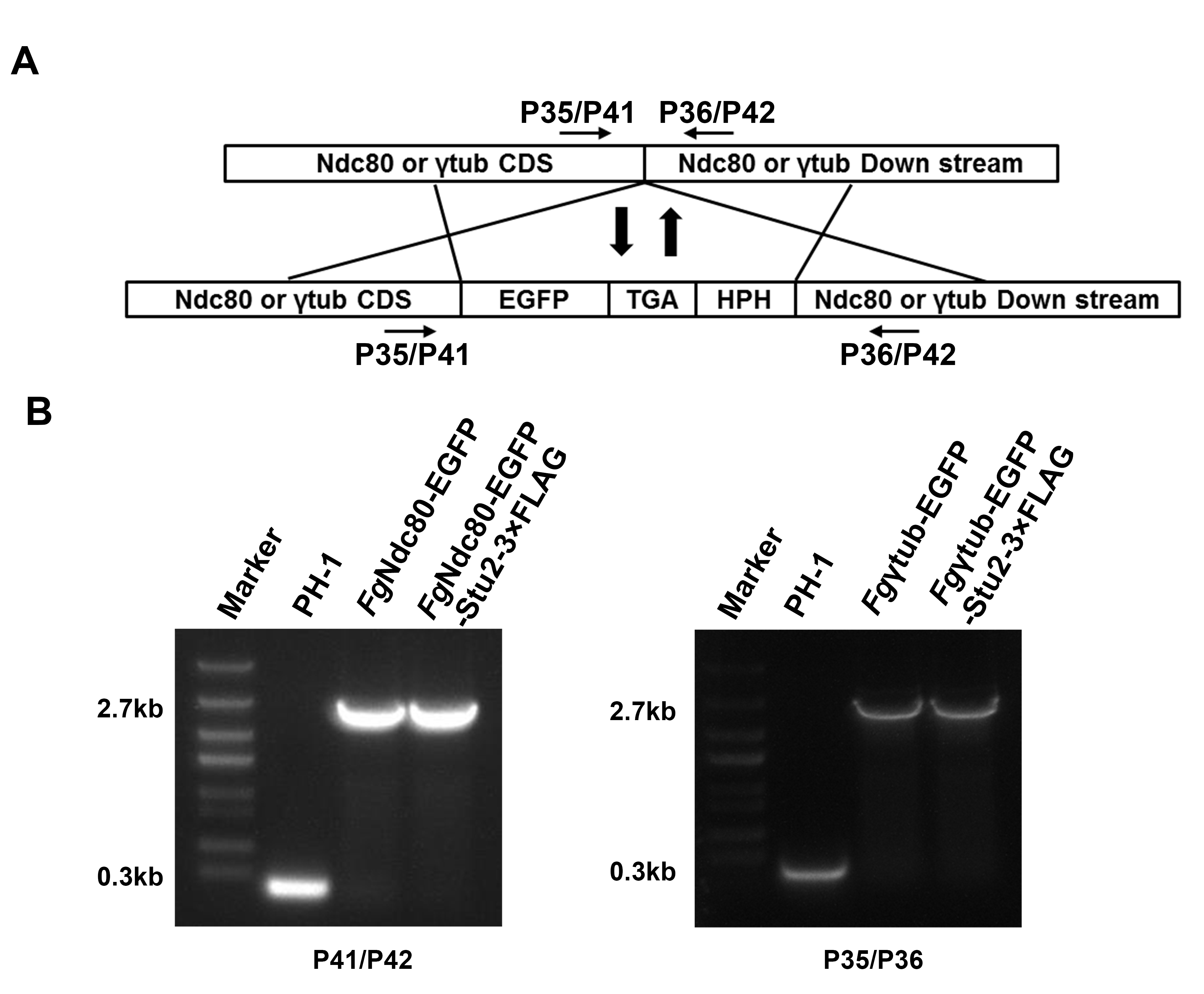

Supplement: Supplementary Figure 5 | — Construction of the Fgγ-tubulin-EGFP and FgNdc80-EGFP strains. (A) Schematic of vector construction. (B) Polymerase chain reaction (PCR) identification of Fgγ-tubulin-EGFP and FgNdc80-EGFP strains. a 2.7 kb fragment was amplified by primers P35/P36 indicates that the EGFP-HPH fragment was successfully inserted at 3′-terminal of Fgγ-tubulin coding sequence. a 2.7 kb fragment was amplified by primers P41/P42 indicates that the EGFP-HPH fragment was successfully inserted at 3′-terminal of presumed FgNdc80 coding sequence. [file Image_5.TIF]

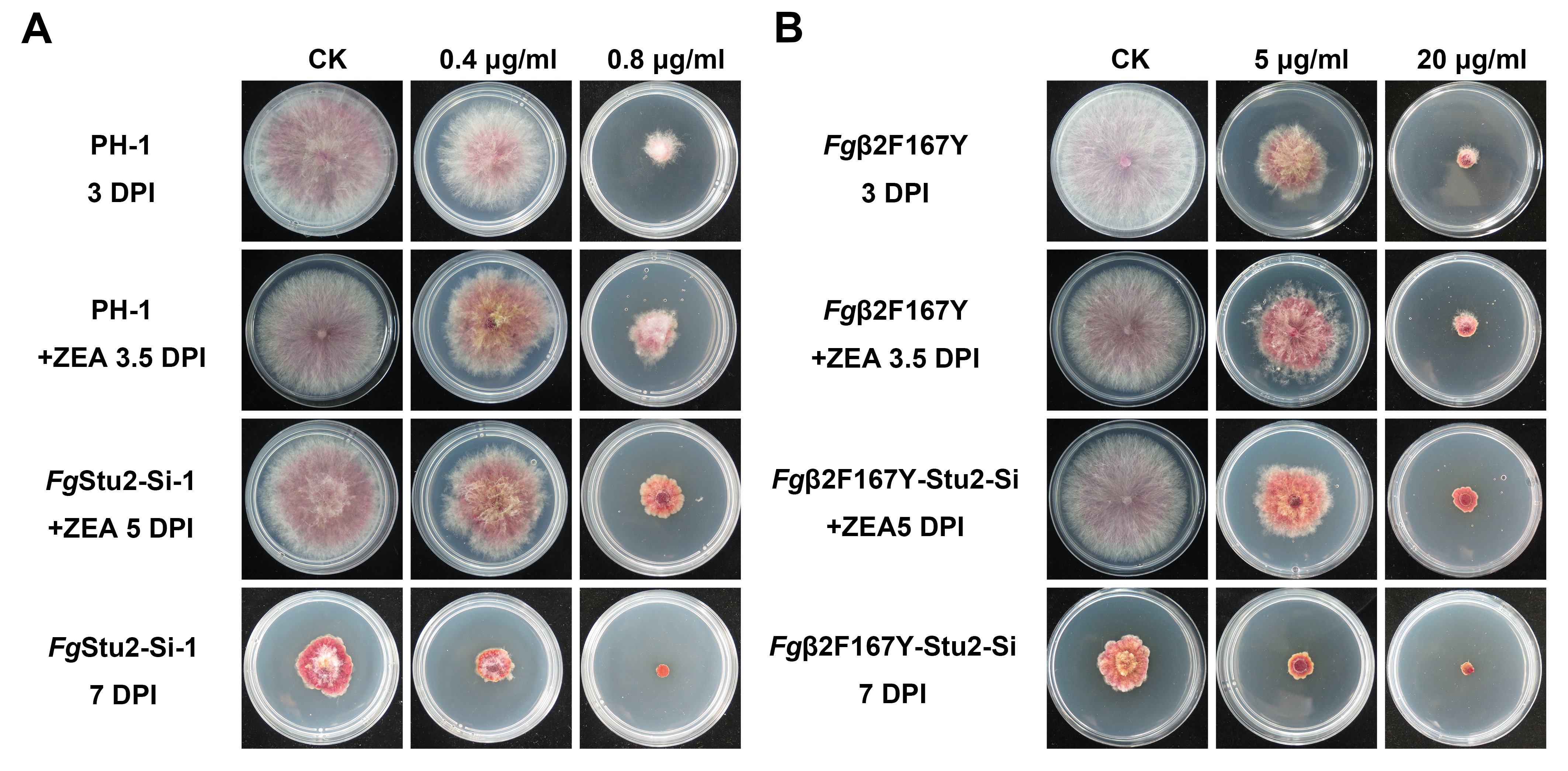

Supplement: Supplementary Figure 6 | — MBC Sensitivity of wild-type strain PH-1, Fgβ 2F167Y, and FgStu2-Si mutants. (A) MBC Sensitivity of wild-type strain PH-1 and FgStu2-Si-1 on PDA plates with or without 30 μM zearalenone (ZEA). (B) MBC Sensitivity of Fgβ2F167Y and F167Y-Stu2-Si on PDA plates with or without 30 μM ZEA. Plugs were transferred from the margins of 3-day old PDA colonies to fresh PDA plants containing different concentrations of MBC. The timing for photography was based on the growth rates of each strain. [file Image_6.TIF]
