# Supplementary material for: The Dis1/Stu2/XMAP215 Family Gene FgStu2 Is Involved in Vegetative Growth, Morphology, Sexual and Asexual Reproduction, Pathogenicity and DON Production of Fusarium graminearum
Source: Front Microbiol. 2020 Nov 20;11:545015. doi: 10.3389/fmicb.2020.545015 (PMC7714731; doi:10.3389/fmicb.2020.545015)
Supplement: Supplementary Table 1 | — Primers used in this study. [file Table_1.DOCX]

**Table S1**. Primers used in this study.

| Primer | Use | Sequence (5' to 3') |
| --- | --- | --- |
| P1 | Amplify a 1.0 kb upstream fragment of *FgStu2* gene | GGGTTGAAGTCACCGAAGTC |
| P2 |  | CAATATCATCTTCTGTCGACCTAGTAAAAGAAACCGATAA |
| P3 | Amplify a 1.1 kb G418 resistant gene fragment used for constructing *FgStu2*-Si strain | TTATCGGTTTCTTTTACTAGGTCGACAGAAGATGATATTG |
| P4 |  | TCAGAAGAACTCGTCAAGAA |
| P5 | Amplify a 0.8 kb fragment of *ZEAR* promoter | TTCTTGACGAGTTCTTCTGACATGCCCTGGCGTTGAAGTT |
| P6 |  | AAATCTTCTTCTTCGGCCATGGTTACTTTCGTTCTCTCTG |
| P7 | Amplify a 1.0 kb opening reading frame of *FgStu2* gene | CAGAGAGAACGAAAGTAACCATGGCCGAAGAAGAAGATTT |
| P8 |  | TCGCGAGAAGCTCGATACAG |
| P9 | Identification of *FgStu2* gene promoter replacement cassette at left junction | AGAGCCATTATCACGTGTGT |
| P10 |  | CTGCGTGCAATCCATCTTGTT |
| P11 | Identification of opening reading frame of *FgStu2* | CGATGTTATACACGAAGCGT |
| P12 |  | ATTGAGTCTCTAGCCTGGTC |
| P13 | Identification of *FgStu2* gene promoter replacement cassette at right junction | GGGCGAGATCTCTAACGATA |
| P14 |  | TTGTCCTTAGGCAGCGAATC |
| P15 | Amplify a 0.6 kb probe fragment used for southern blot verification of *Fg*Stu2-Si mutant | GAAGAAGGCTGCGGTAGCCG |
| P16 |  | GACCAGGCTTGGTAAGCTTG |
| P17 | Amplify a 0.7 kb opening reading frame of RFP from pRFP plasmid for fusing with *Fg*Stu2 coding sequence | CAGATCTTGGCTTTCGTAGGAACCCAATCTTCAATGGCCTCCTCCGAGGACGT |
| P18 |  | GGCGCCGGTGGAGTGGCGG |
| P19 | Amplify a 2.6 kb opening reading frame of *Fg*Stu2 from genomic DNA of PH-1 for fusing with RFP | CCGCCACTCCACCGGCGCCGGAGGTAGTGGAGGTATGGCCGAAGAAGAAGATTT |
| P20 |  | CACCACCCCGGTGAACAGCTCCTCGCCCTTGCTCACCTAAGGTCGAGCGAGTCCTT |
| P21 | Amplify a 1.0 kb 3' terminal opening reading frame of *Fg*Stu2 (forward primer) | CCTGGTCTCGGTGGATTG |
| P22 | Amplify a 1.0 kb 3' terminal opening reading frame of *Fg*Stu2 containing 1xFlag tag (Reverse primer) | CTTGTCGTCGTCGTCCTTGTAGTCAGGTCGAGCGAGTCCTTGCT |
| P23 | Amplify a 1.0 kb 3' terminal opening reading frame of *FgStu2* containing 2xFlag tag (Reverse primer) | CTTATCATCATCATCCTTGTAATCCTTGTCGTCGTCGTCCTTGTAGTC |
| P24 | Amplify a 1.0 kb 3' terminal opening reading frame of *Fg*Stu2 containing 3xFlag tag (Reverse primer) | CTACTTGTCGTCGTCGTCCTTGTAGTCCTTATCATCATCATCCTTGTAATC |
| P25 | Amplify a 1.1kb fragment of G418 resistant gene used for constructing *Fg*Stu2-3×Flag strain | GACTACAAGGACGACGACGACAAGTAGGTCGACAGAAGATGATATTG |
| P26 |  | ACAATTTTTTCTTGACAATTTCAGAAGAACTCGTCAAGAA |
| P27 | Amplify a 1.1 kb downstream fragment of *Fg*Stu2 gene used for constructing *Fg*Stu2-3×Flag strain | TTCTTGACGAGTTCTTCTGAAATTGTCAAGAAAAAATTGT |
| P28 |  | CGATGCCATCACCGCTCAAG |
| P29 | Identification of G418 insertion at 3' terminal of *Fg*Stu2 CDS | TAGCAAGCAGCATGTCTGAA |
| P30 |  | GGTGGCACAAGTTCTAACGC |
| P31 | Amplify a 1.0 kb opening reading frame of 3' terminal of *Fg*γ-tubulin gene | ACAGTATCCTATCCATGCGC |
| P32 |  | TCCTCGCCCTTGCTCACCATCCCTACTCGTCGATCTGTCT |
| P33 | Amplify a 0.9 kb downstream fragment of *Fg*γ-tubulin gene | TACGCAAACCGCCTCTCCCCGGGGCAGGGCACTACGGATC |
| P34 |  | TGCCAGTCAGAGGCAGTA |
| P35 | Identification of EGFP-HPH (hygromycin resistant gene) insertion at 3' terminal of *Fg*γ-tubulin gene | GAGTTTGACGAGGCACGGC |
| P36 |  | CTCTAATACGCCTACAAGCA |
| P37 | Amplify a 1.0 kb 3' terminal opening reading frame of *Fg*Ndc80 gene | ACGGTCCGAGAAGTACGAGA |
| P38 |  | TCCTCGCCCTTGCTCACCATCATATCCATTCGTTGAGTGT |
| P39 | Amplify a 1.0 kb downstream fragment of *Fg*Ndc80 gene | TACGCAAACCGCCTCTCCCCAGCAATCTTTCAAGACGCTT |
| P40 |  | GCTAAGCATGGACGATGCAC |
| P41 | Identification of EGFP-HPH (hygromycin resistant gene) insertion at 3' terminal of presumed *Fg*Ndc80 gene | AAGATCCATGTCCAAAAGAG |
| P42 |  | GGACCAGCCAGACGCAACTT |
| P41 | Amplify a 0.7 kb EGFP fragment | ATGGTGAGCAAGGGCGAGGA |
| P42 |  | TTACTTGTACAGCTCGTCCA |
| P41 | Amplify a 1.7kb fragment of hygromycin resistant gene | GGGAGCTGTTGGCTGGCTGG |
| P42 |  | GGGGAGAGGCGGTTTGCGTA |
| P43 | Amplify the *Fg*Stu2 gene for quantitative real-time polymerase chain reaction | GAAGCGCACGACCCTATAAT |
| P44 |  | ACCGTTACCTGTTGGTTGAG |
| P45 | Amplify the reference gene *Fg*Actin for quantitative real-time polymerase chain reaction | ATCCACGTCACCACTTTCAA |
| P46 |  | TGCTTGGAGATCCACATTTG |
| P47 | Amplify the reference gene *Fg*Tri5 gene for quantitative real-time polymerase chain reaction | CACTTGTCAACGAGCACTTTC |
| P48 |  | TGCTCAATCCAACATCCCTC |
| P49 | Amplify the reference gene *Fg*Tri6 gene for quantitative real-time polymerase chain reaction | GACTGTTGGACGCAGTGCCA |
| P50 |  | CGACTTCTTGCAGGTCTTGAGC |
| P51 | Amplify a 1.7kb fragment of FgTri1 gene CDS for construction of PDL2-*Fg*Tri1-EGFP plasmid | CAGATCTTGGCTTTCGTAGGAACCCAATCTTCAATGGCTCTCATCACCAGTTT |
| P52 |  | CACCACCCCGGTGAACAGCTCCTCGCCCTTGCTCACGTCATCCTGTACCAATTCCA |
